# The therapeutic window of intravenous immunoglobulin (IVIG) and its correlation with clinical outcomes in Kawasaki disease: a systematic review and meta-analysis

**DOI:** 10.1186/s13052-023-01451-6

**Published:** 2023-04-11

**Authors:** Zheng Li, Jianghui Cai, Jing Lu, Mingju Wang, Chenmei Yang, Zheng Zeng, Qian Tang, Jianhong Li, Wen Tang, Huiling Luo, Gaofeng Pan, Xingmao Zeng

**Affiliations:** 1 Department of Neurology, The Second Clinical Medical College, Geriatric Diseases Institute of Chengdu/Cancer Prevention and Treatment Institute of Chengdu, Chengdu Fifth People’s Hospital, Affiliated Fifth People’s Hospital of Chengdu University of Traditional Chinese Medicine), Chengdu, 611130 China; 2 Department of Pharmacy, School of Medicine, Chengdu Women’s and Children’s Central Hospital, University of Electronic Science and Technology of China, Chengdu, 611731 China; 3 Department of Neurology, The Second Clinical Medical College, Geriatric Diseases Institute of Chengdu/Cancer Prevention and Treatment Institute of Chengdu, Chengdu Fifth People’s Hospital, Affiliated Fifth People’s Hospital of Chengdu University of Traditional Chinese Medicine), No.33 Mashi Street, Wenjiang District, Chengdu city, 611100 China

**Keywords:** Kawasaki disease, IVIG, Intravenous immunoglobulin, IVIG resistance, CALs

## Abstract

**Background:**

The optimal therapeutic window to start intravenous immunoglobulin (IVIG) for Kawasaki disease (KD) is highly debatable. We aimed to summarize the existing literature to evaluate the therapeutic window of IVIG treatment and its correlation with clinical outcomes in KD patients.

**Methods:**

We searched the databases from inception to August 26, 2022, without language restrictions. The primary outcomes were initial IVIG resistance and coronary artery lesions (CALs) in acute phase. Secondary outcome was CALs during 1–2 months of follow-up.

**Results:**

27 studies involving 41,139 patients were included in this study. Very low-quality evidence showed that the earlier IVIG treatment within 4 days had a higher IVIG-resistance rate (RR, 1.80; 95% CI, 1.50–2.15; P < .00001; I^2^ = 75%) than the late treatment. Very low-quality evidence showed that IVIG treatment for more than 7 days was associated with a higher risk of CALs in acute phase(RR, 0.57; 95% CI, 0.40–0.80; P = .001; I^2^ = 76%). There was a lower risk of CALs during 1–2 months follow-up for those who started IVIG administration within 10 days from the onset.

**Conclusions:**

Overall, IVIG treatment within 7 days of illness seems to be the optimal therapeutic window of IVIG. IVIG treatment within 7 days is found to be effective for reducing the risk of coronary artery lesions and cardiac sequelae in KD patients. The early IVIG treatment within 4 days should be vigilant for the IVIG resistance although large multi-center randomized trials with well design are needed.

**Supplementary Information:**

The online version contains supplementary material available at 10.1186/s13052-023-01451-6.

## Background

Kawasaki disease (KD) is an acute, febrile vasculitis of unknown aetiology that primarily affecting children < 5 years of age [1]. Major classical features of KD include fever, conjunctivitis, erythema of the lips, oral mucosa, changes in extremities, rash, and cervical lymphadenopathy. KD has been reported in more than 60 countries worldwide since first described in Japan in 1974 [2]. The incidence of KD is increasing, and the highest relative risk is in Asian children, especially in eastern Asia [3]. KD has surpassed acute rheumatic fever as the most common cause of acquired heart disease in developed countries [4].

The most common sequel caused by KD is coronary artery lesions (CALs), including coronary artery dilatation and coronary artery aneurysm (CAA) [5]. CAA can develop in around 25% of untreated KD children [1]. Intravenous immunoglobulin (IVIG) in conjunction with aspirin remains the first-line drug therapy for KD patients [1]. The mortality rates of KD have been markedly decreased since the IVIG therapy was introduced in 1983 [6], and the incidence of CALs in those treated appropriately is dropped to 4% [1]. However, several latest clinical guidelines on the optimal timing of IVIG administration and if IVIG can be given earlier remain inconclusive [1, 7, 8]. Some previous retrospective studies reported that IVIG treatment within 5 days of illness reduces the risk of cardiac sequelae, suggesting the efficacy of the earlier initiation of IVIG treatment [9–11]. On the contrary, other studies reported that IVIG use at ≤ 4 days of illness had no benefit in preventing coronary complications but was instead associated with increased IVIG resistance [12, 13]. Therefore, the association between the earlier timing of IVIG administration of disease onset and the risk for IVIG unresponsiveness remains controversial and debatable [14].

Given this background, there is a need to qualitatively and critically appraise available evidence and evaluate where gaps exist. Thus, we conducted a systematic review and meta-analysis to investigate the optimal timing of IVIG administration in KD patients by comparing the clinical outcomes of early and conventional IVIG treatment.

## Methods

This systematic review and meta-analysis aimed to critically appraise the therapeutic window of IVIG treatment and its correlation with clinical outcomes in KD patients. We performed this systematic review based on Preferred Reporting Items for Systematic Reviews and Meta-analyses (PRISMA) guidelines [15]. The protocol was prospectively registered in the International Prospective Register of Systematic Reviews (PROSPERO; registration number: CRD42022356138).

## Data sources and search strategy

We systematically searched databases, including PubMed (MEDLINE), Cochrane Library (CENTRAL), Embase, Web of Science, and Chinese Biomedical Literature database (CBM) from database inception to August 26, 2022, without language restrictions. An e-mail alert was received weekly based on a previously developed search strategy saved in PubMed for any new potential studies. For completed and ongoing trials, we also searched trial registries, including ClinicalTrials.gov and the international Clinical Trial registry platform (ICTRP). Finally, we hand-searched reference lists of identified articles for further eligible trials. A search strategy was built based on MeSH terms and free-text words based on the following terms: Mucocutaneous Lymph Node Syndrome, Kawasaki disease, intravenous immunoglobulin, and gamma-Globulins. A detailed search strategy can be seen in ***Supplementary Material, Appendix S1***.

## Eligibility criteria

Studies that met all of the following criteria were included: (a) Patients with KD (diagnosed using any recognized diagnostic criteria) undergoing early (≤ day 4), conventional (days 5–10) or late (>day 10) IVIG treatment; (b) study design: randomized controlled trials (RCTs), cross-sectional study, cohort, case-control; (c) with adequate data on necessary basic characteristics and outcomes.

Exclusion criteria were as follows: (a) KD patients complicated with severe infection, allergy, autoimmune diseases, or collagen disease; (b) case reports, reviews, guidelines, opinions, editorials, letters, animal studies, and comments.

## Study selection

Study selection was managed using Covidence software (https://www.covidence.org/). Two independent reviewers (ZZ and XMZ) evaluated articles for potential inclusion by screening titles and abstracts. The full texts of those identified as being relevant would then be assessed to determine eligibility for final inclusion. The results would be discussed between each assessment to reach a consensus on the interpretation of the inclusion criteria. Any disagreements regarding study eligibility would be resolved by consensus, and a third reviewer (WT) was consulted if necessary. If the information required to assess eligibility were unavailable or unclear, the relevant study authors would be contacted for clarification. Duplicate publications were identified and removed using EndNote software version X7 (Clarivate Analytics). The identified publications were analyzed using criteria based on largest sample size, the maximum correspondence with the inclusion criteria, and a minimal risk of bias.

## Data extraction

Two authors (JL and CMY) independently extracted data in duplicate using a predefined data extraction form (Excel, Microsoft Corporation, USA). We did a thorough pilot test before the formal data collection to ensure consistency in the data extraction process and that all necessary information was collected. The following data were extracted from the studies selected for inclusion, as follows: (a) general characteristics of the study (author names, publication date, study design, study period, setting, funding and country); (b) patient demographic features (including sample size, groups, age, gender, the proportion of complete KD cases, recurrent cases and patients who received IVIG treatment); (c) outcome measures and analyses; (d) IVIG treatment protocol and definitions of IVIG resistance.

The primary outcomes were the incidence of initial IVIG resistance and CALs in the acute phase. The secondary outcome was CALs during 1–2 months follow-up.

## Risk of bias and grade certainty assessment

Two reviewers (GFP and HLL) independently assessed the risk of bias based on articles and protocols. Any discrepancies were solved by discussion and intervention of a third reviewer (QT) whenever necessary. We used the Risk of Bias Tool 2.0 (RoB 2.0) developed by the Cochrane Collaboration for randomized trials [16]. This tool evaluates five domains of bias: randomization process, deviations from intended interventions, missing outcome data, measurement of the outcome, and selection of the reported results. The RoB 2.0 for each of the 5 domains is described as low, some concerns, or high. The risk of bias was assessed using the Newcastle-Ottawa scale (NOS) for observational cohort and case-control studies [17]. There were three grouping items as follows: selection, comparability, and exposure/outcomes. A study can be awarded a maximum of one star for each numbered item within the selection and outcome categories. A maximum of two stars can be given for comparability. More stars are equalling lower risk. The NOS scale awards a maximum of nine stars to each study. The summary score given to studies can be categorized as poor (0–3 points), fair (4–6 points), or high (≥ 7 points) quality. The quality of evidence of the included studies was assessed according to The Grading of Recommendations Assessment, Development and Evaluation (GRADEpro Guideline Development Tool, available online at gradepro.org) [18].

### Statistical analysis

Results were assessed using forest plots and presented as RRs for the binary outcomes. Heterogeneity was assessed by I² tests, and substantial heterogeneity was defined as I² greater than 50%. An inverse variance, fixed-effects method was used if I² was less than 50%, and a random-effects method was used if I² was 50% or greater. We performed subgroup analyses based on research types, study location and type of CALs (coronary artery dilation or CAA). Sensitivity analysis was performed by evaluating the pooled estimate after omitting a study each time. Publication bias was assessed by visually inspecting a funnel plot and quantified by the Egger test. Statistical analyses were conducted using ReviewManager version 5.2 (RevMan; Copenhagen: The Nordic Cochrane Center, the Cochrane Collaboration, 2014, London, UK) and Stata statistical software version 14.0 (StataCorp, College Station, Texas) software. A two-sided p-value less than 0.05 was set as the threshold for statistical significance.

## Results

**Literature search and study selection**.

**Fig. 1:**
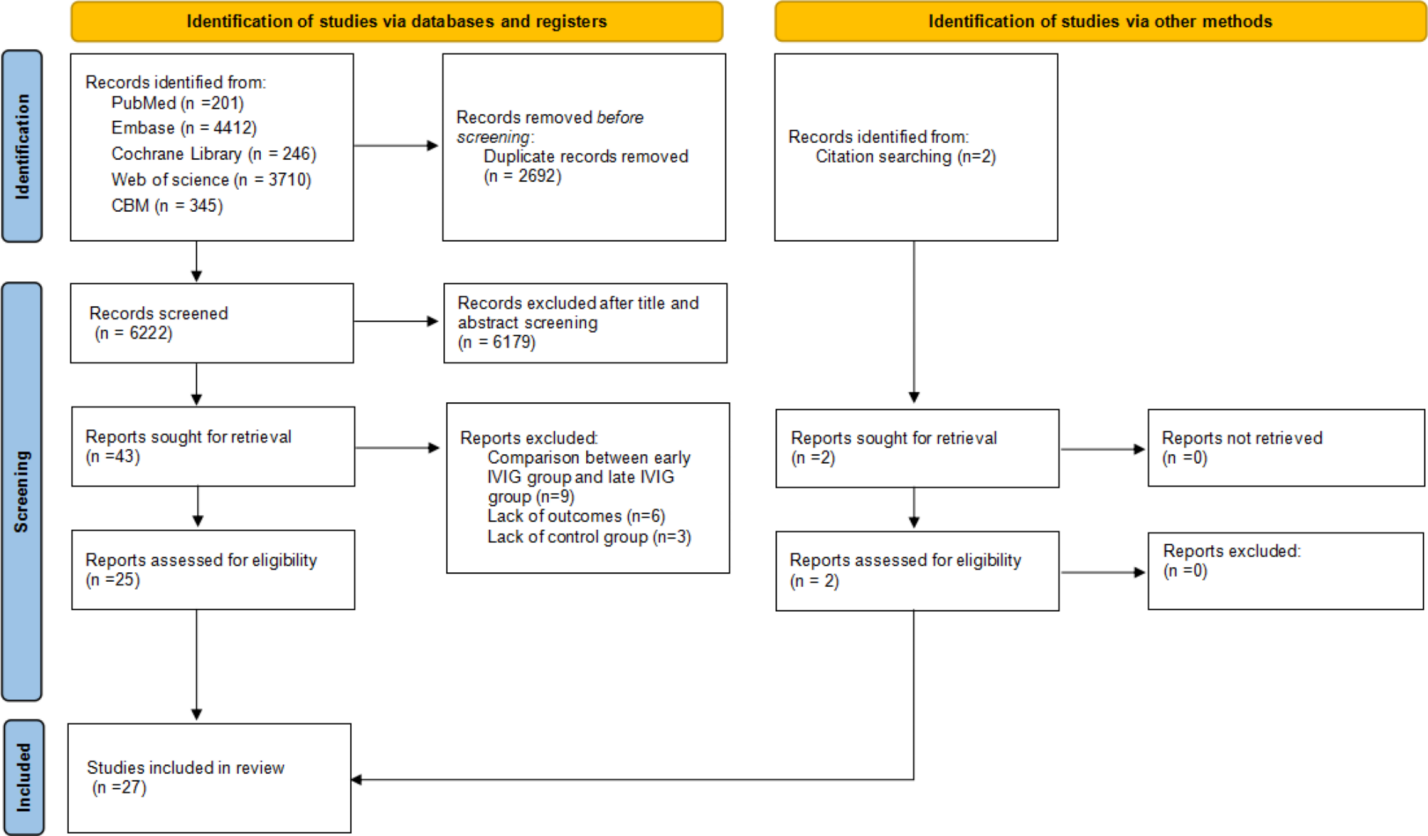
PRISMA Flow Diagram

We identified 8914 articles by the initial database search, including 201 from Pubmed, 4412 from Embase, 246 from the Cochrane database, 3710 from Web of Science, and 345 from CBM. After removing duplicates (n = 2692) and articles based on screening of the title and abstract screening (n = 6179), 43 full-text articles were assessed for eligibility. Furthermore, two articles were identified through citation searching. After screening of full texts, 18 articles that did not meet the inclusion criteria were excluded (reasons for exclusion are summarized in ***Supplementary Material, Appendix S2***). A total of 27 studies [9–13, 19–40] met the inclusion criteria and were analyzed for the systemic review and meta-analysis (Fig. 1).

## Characteristics of the included studies

The included studies were all published from 2002 to 2022 and encompassed 41,139 patients. These studies originated from 7 countries, with sample sizes ranging from 31 to 20,845. Studies were published in English and Chinese. Most of the studies were observational. Eighteen (66.7%) were cohort studies [9, 10, 12, 13, 19–32], three (11.1%) were case-control studies [11, 33, 34], and six (22.2%) were RCTs [35–40]. The included articles showed that the clinical outcomes differed among four categories (start of IVIG therapy up to 4 days, 5 to 7 days, 8 to 10 days, and more than 10 days). The heterogeneity might be due to the participant characteristics, IVIG treatment protocol, the definition of IVIG resistance and the definition of CALs. The IVIG treatment protocol and specific definitions used for IVIG resistance were at the individual study investigators’ discretion (***Supplementary Material, Appendix S3***). Details of the included studies are described in Table 1.

**Table 1:** Characteristics of the included studies

Author	Year	Study design	Study period	Setting	Funding	Country	sample size	Groups	Sex [male, %]	Age[yr, median (IQR) or mean (SD)]	Complete KD cases[%]	Recurrent cases[%]	Received IVIG[%]	Outcomes
Cai	2022	Cohort	2018.01- 2020.12	Chengdu Women’s and Children’s Central Hospital	No	China	965	Early group (day 1–4); Conventional group (day 5–7); Conventional group (day 8–10); Late group (after day 10).	60.5	2.3 (1.6)	91.5	0	100.0	①②③④
Li	2021	Cohort	2015.01- 2017.12	Guangzhou Women and Children’s Medical Center、the First Affiliated Hospital of Sun Yat-sen University	Yes	China	1281	Group 1 (≤ 4days); Group 2 (5–7 days); Group 3 (8-10days); Group 4 (> 10days).	63.7	1.0 (0.9-3.0)	NA	NA	100.0	①②③④
Yazdi	2021	Cohort	2012.01- 2015.12	Imam Khomeini Hospital	No	Iran	31	Early detection group (≤ 10days); Late detection group (>10days).	54.8	3.6 (0.2)	100.0	NA	100.0	②
Ha	2020	Cohort	2008.01- 2014.12	Korea University Medical Center	No	Korea	555	Early (≤ 3 days); Usual (4–5 days); Late (≥ 6 days).	57.8	NA	NA	NA	100.0	①②③
Shiozawa	2018	Cohort	2006.08- 2013.01	Eight hospitals in Japan	No	Japan	339	Early treatment group (by day 4 of illness); Conventional treatment group (on day 5).	36.6	1.8	100.0	0	100.0	①②
Kuwabara	2018	Cohort	2011.01- 2012.12	22nd nationwide survey of KD in Japan	Yes	Japan	20,845	Early (4 days or less); Conventional (5 to 7 days); Late (8 to 10 days).	42.2	NA	84.8	0	100.0	①②
Downie	2017	Cohort	1990.01- 2013.12	Hospital for Sick Children	Yes	Canada	1269	Prompt treatment (≤ 10 days); Delayed treatment (>10 days).	61.5	(1.5-6.0)	73.0	NA	100.0	①②③
Mohammadzadeh	2016	Cohort	2006–2011	Amirkola Children’s Hospital	Yes	Iran	100	Early (within 10 days); Late (> 10 days).	61.0	2.8 (2.6)	77.0	NA	100.0	②
Chen	2015	Cohort	2008.01- 2012.12	50 hospitals in Shanghai	Yes	China	2304	Early (< 5 days); Conventional (5 to 10 days); Late (> 10 days).	66.2	2.3 (0.1–11.7)	72.9	0.9	94.3	②
Bal	2014	Cohort	1999.01- 2011.12	Jersey Shore University Medical Center	No	USA	106	Early (within 10 days); Late (> 10 days).	63.2	(0–18.0)	91.5	NA	100.0	①②③④
Wang	2014	Cohort	2010.02- 2013.08	Logistic University of PAPF	No	China	60	Observation group (>5days); Control group (applied immediately).	51.7	(0. 5–10.0)	NA	NA	100.0	①
Callinan	2012	Cohort	2000–2009	US Centers for Disease Control and Prevention’s national KS surveillance system	Yes	USA	2056	Before illness day 5; After illness day 5.	59.6	< 18	98.0	1.4	98.0	②
Sittiwangkul	2011	Cohort	2000–2008	Chiang Mai University Hospital	No	Thailand	170	Group I (≤ 10 days of fever); Group II (>10 days of fever).	60.0	21.8 (1.4)	74.7	NA	91.2	①②
Du	2009	Cohort	2000–2004	45 hospitals in Beijing	Yes	China	1052	Early (1-4days); Conventional (5-9days); Late (≥ 10days).	64.6	(0.2-l3.8)	NA	NA	100.0	①②③④
Muta	2004	Cohort	1997-1998 1999-2000	The database from nationwide surveys in Japan	Yes	Japan	8751	Early group (1-4days); Conventional group (5-9days).	59.1	(0.1–21.3)	85.9	0	100.0	①②
Li	2006	Cohort	1998.01- 2005.03	Affiliated Taihe Hospital of Yunyang Medical College	No	China	41	Group A (≤ 5days); Group B (6-10days); Group C (>10days).	56.1	(0.5-6.0)	NA	NA	100.0	①②④
Hsieh	2004	Cohort	1993–2003	Veterans General Hospital-Kaohsiung	Yes	China	162	Before illness ady 5; After illness day 5.	61.7	1.79 (1.58)	NA	NA	100.0	①②
Nomura	2002	Cohort	1989.01- 1998.12	Kagoshima City Medical Association Hospital	No	Japan	125	Group A (from the second to the fourth day); Group B (from the fifth day to ninth).	56.8	(0.2–11.3)	100.0	NA	100.0	①②③④
Muta	2012	Case- control	2007.01- 2008.12	The database of the 20th nationwide survey of KD in Japan	Yes	Japan	150	Late IVIG (Days 11–20); Conventional IVIG (Days 4–8).	50.0	(0.2–7.9)	91.3	NA	100.0	①②③④
Fong	2004	Case- control	1994–1999	Princess Margaret Hospital	No	China	81	Case group (before day 5 of fever); Control group (on day 5 of fever or after).	61.7	NA	NA	NA	100.0	①②③
Tse	2002	Case- control	1987–1999	Hospital for Sick Children	Yes	Canada	178	Case group (≤ 5 days of fever); Control group (between 6 and 9 days of fever).	62.9	NA	100.0	NA	100.0	①②③④
Kong	2021	RCT	2018.01- 2019.12	Huainan Women’s & Children’s Hospital	No	China	61	Observation group (5-7days); Control group (8-10days).	55.7	(0.4-6.0)	NA	NA	100.0	①②③④
Wu	2019	RCT	2015.05- 2018.10	Mianzhu People’s Hospital	No	China	123	Observation group (1-5days); Control group1 (5-10days); Control group1 (>10days).	56.9	3.46 (0.38)	100.0	NA	100.0	①②③④
Xiong	2019	RCT	2015.05- 2018.02	The First People’s Hospital of Guangshui City	No	China	70	Observation group (5-10days); Control group (>10days).	60.0	(0.6–11)	NA	NA	100.0	②
Shao	2018	RCT	2011.09- 2017.06	Wenling First People’s Hospital	No	China	64	Observation group (5-9days); Control group (10-14days).	59.4	NA	0	NA	100.0	②④
An	2017	RCT	2015.01- 2015.12	The Affiliated Hospital of Hebei University	No	China	110	Early (1-5days); Conventional (5-10days); Late (≥ 10days).	53.6	(0.4–12)	NA	0	100.0	①②
Li	2013	RCT	2009.01- 2010.10	Shuangliu Second People’s Hospital	No	China	90	Group I (1-4days); Group II (5-9days); Group III (9-13days).	48.9	(0.6–10)	NA	NA	100.0	①②

★Data are presented as mean (standard deviation) or median (IQR) or number (%);

★①: IVIG resistance; ②: CALs in the acute phase; ③: CALs during 1–2 months follow-up; ④: Coronary artery dilation;

★CALs = coronary artery lesions; CAAs = coronary artery aneurysms; KD = Kawasaki disease; IVIG = intravenous immunoglobulin; RCT = randomized controlled trial; NA = not applicable

## Assessments of risk of bias

All observational studies were of fair-to-high quality, and all randomized studies were at some concerns. The results for each quality assessment by the study are presented in ***Supplementary Material, Appendix S4*** and ***Supplementary Material, Appendix S5***.

## Certainty of the evidence

We rated the certainty of the evidence of primary outcomes using by GRADE approach. The analysis results showed that the overall quality of evidence was very low, mainly due to more observational designs we included and inconsistency (I^2^ > 50%). The outcomes and assessments were presented as a summary of findings in ***Supplementary Material, Appendix S6***.

## Primary outcomes

### Initial IVIG resistance

**Fig. 2:**
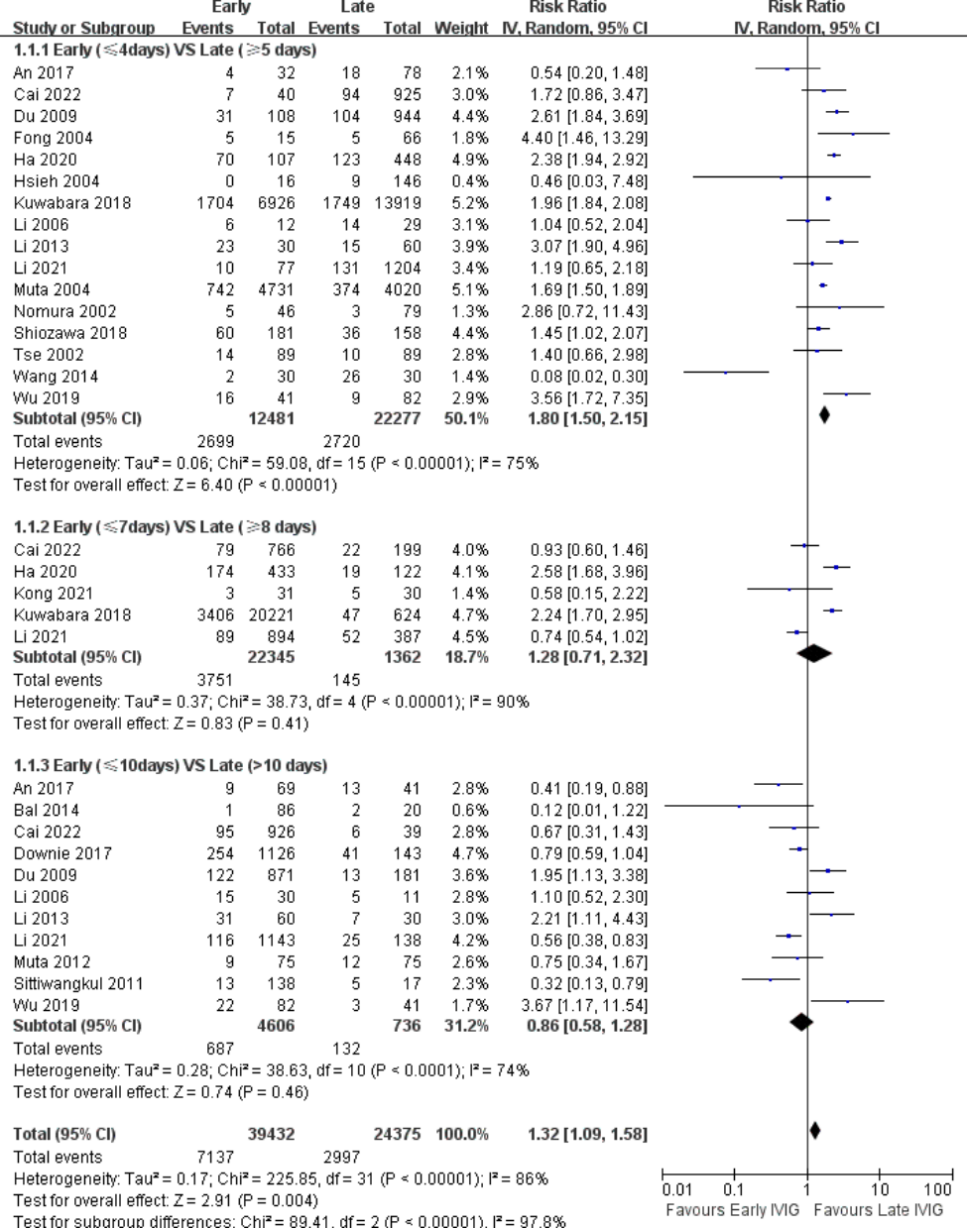
Forest Plot of risk ratios for initial IVIG resistance for Early IVIG VS. Late IVIG.

Due to a lack of data, we did not include repeated IVIG resistance in our analysis. Of the 27 studies, 21 studies [9–13, 19, 21, 23–25, 28, 29, 31–33, 35–40] including 36,499 participants reported initial IVIG resistance, and 5764 had IVIG resistance (15.8%). Very low-quality evidence showed that, using day 4 of fever as the cut-off point, the early group ( ≦ 4 days) had a higher initial IVIG-resistance (RR, 1.80; 95% CI, 1.50–2.15; P < .00001; I^2^ = 75%) than the late group (>4 days), with 21.6% (2699 of 12,481) in the early group VS 12.2% (2720 of 22,277) in the late group (Fig. 2). Moreover, at the 7-day (RR, 1.28; 95% CI, 0.71–2.32; P = .41; I^2^ = 90%) and 10-day (RR, 0.86; 95% CI, 0.58–1.28; P = .46; I^2^ = 74%) cut-off point, the prevalence of IVIG resistance had no significant difference between the groups (Fig. 2).

## The occurrence of CALs in the acute phase

**Fig. 3:**
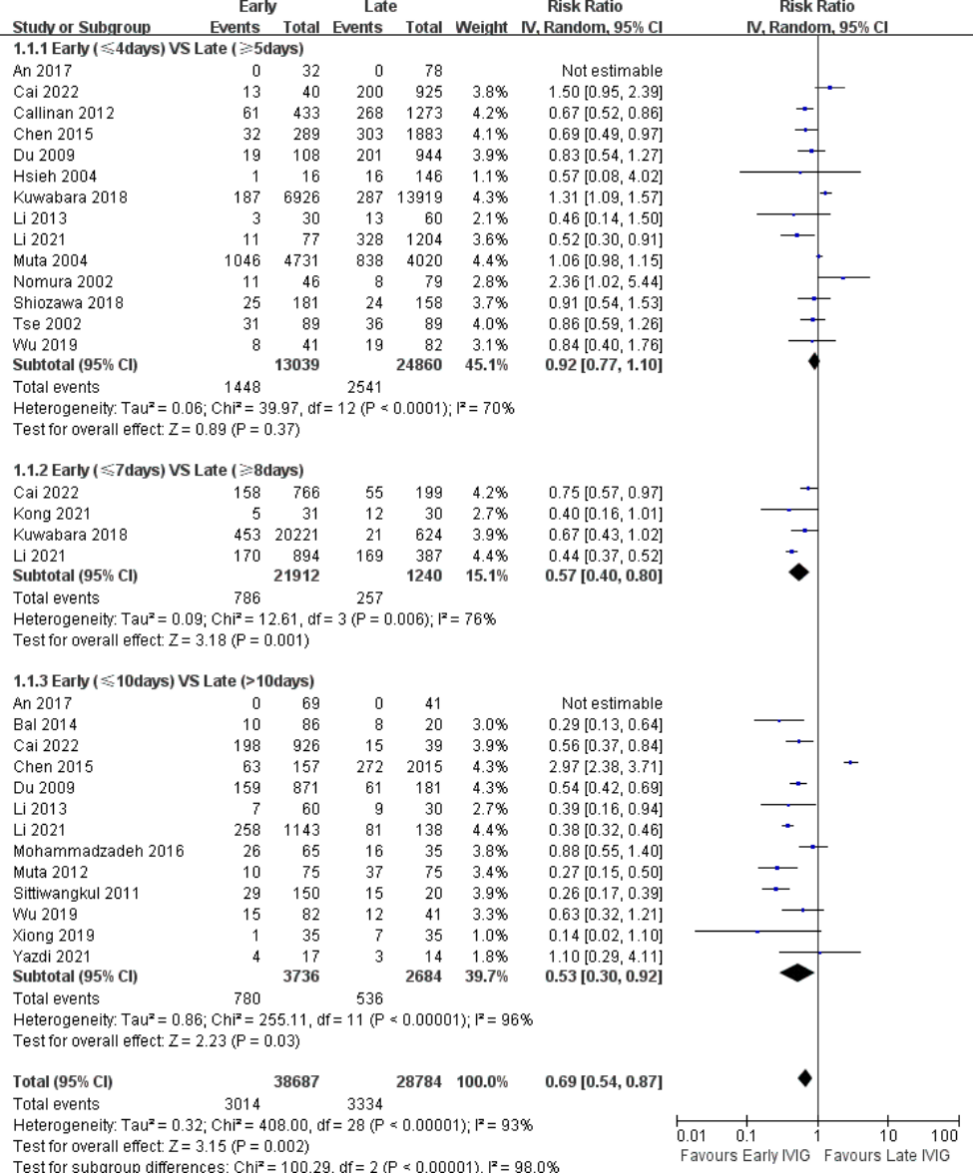
Forest Plot of risk ratios for CALs in the acute phase for Early IVIG VS. Late IVIG.

21 studies [9–13, 19, 21, 23–25, 28, 29, 31–33, 35–40] including 38,587 participants investigated the occurrence of CALs in the acute phase, and 4172 had CALs (10.8%). Very low-quality evidence showed that using day 4 of fever as the cut-off point, the two groups of KD patients had a similar likelihood of CALs in the acute phase (RR, 0.92; 95% CI, 0.77–1.10; P = .37; I^2^ = 70%) (Fig. 3). However, at the 7-day (RR, 0.57; 95% CI, 0.40–0.80; P = .001; I^2^ = 76%) and 10-day (RR, 0.53; 95% CI, 0.30–0.92; P = .03; I^2^ = 96%) cut-off point, the rate of CALs in the late group was significantly higher than the early group (Fig. 3).

## Secondary outcomes

### CALs during 1–2 months follow-up

13 studies [9, 11, 12, 19–23, 25, 29, 31, 33, 39] reported CALs during 1–2 months follow-up (654 of 6652 [9.8%] vs. 696 of 6995 [9.9%]). The pooled analysis showed that delaying IVIG treatment for more than 10 days was associated with a higher risk of CALs during 1–2 months follow-up than the earlier treatment (OR, 0.49; 95% CI, 0.32–0.76; P = .002; I^2^ = 75%). However, at the 4-day (RR, 1.11; 95% CI, 0.81–1.52; P = .51; I^2^ = 52%) and 7-day (RR, 0.63; 95% CI, 0.23–1.67; P = .35; I^2^ = 86%) cut-off point, there was no difference between the groups (***Supplementary Material, Appendix S7***).

## Sensitivity analysis

We conducted several sensitivity analyses to confirm the robustness of our findings. In the sensitivity analysis, the total findings did not change significantly. All sensitivity analyses indicated that the results of the meta-analysis were robust and the heterogeneity reduced to some extent (***Supplementary Material, Appendix S8*** and ***S9***).

## Subgroup analysis

Subgroup analyses were performed based on research types and the study location (***Supplementary Material, Appendix S10*** and ***S11***). For the subgroup of research types, the pooled analysis of randomized trials, which had a broader confidence interval, smaller sample size and higher heterogeneity (RR, 1.97; 95% CI, 0.76–5.07; P = .16; I^2^ = 81%), showed that early initiation of IVIG treatment within 4 days was not associated with a higher risk of initial IVIG resistance. The pooled analysis from 13 observational studies showed that early IVIG treatment (≤ 4 days ) was associated with a higher risk of initial IVIG resistance (RR, 1.74; 95% CI, 1.45–2.09; P < .00001; I^2^ = 74%), but there was evidence of heterogeneity across the studies. The subgroup analyses of CALs in the acute phase showed similar findings based on research types. There were no significant differences in the occurrence of CALs (≤ 4 days VS > 4 days) in both RCTs (RR, 0.71; 95% CI, 0.38–1.33; P = .28; I^2^ = 0%) and observational studies (RR, 0.94; 95% CI, 0.78–1.14; P = .51; I^2^ = 74%).

For the subgroup of the study location, we found that the studies performed in Japan (RR, 1.79; 95% CI, 1.56–2.05; P < .00001; I^2^ = 60%) and Korea (RR, 2.38; 95% CI, 1.94–2.92; P < .00001) showed an increased risk in the occurrence of initial IVIG resistance with early IVIG treatment within 4 days. Meanwhile, studies conducted in China (RR, 1.43; 95% CI, 0.85–2.42; P = .18; I^2^ = 81%) and Canada (RR, 1.40; 95% CI, 0.66–2.98; P = .38) showed that early IVIG treatment within 4 days is not associated with a higher risk of initial IVIG resistance. The subgroup analyses of CALs in the acute phase showed similar findings based on the study location. There were no significant differences in the incidence of CAL (≤ 4 days VS > 4 days) regardless of the study location (China: RR, 0.79; 95% CI, 0.58–1.08; P = .14; I^2^ = 46%; Japan: RR, 1.17; 95% CI, 0.95–1.44; P = .13; I^2^ = 63%; Canada: RR, 0.86; 95% CI, 0.59–1.26; P = .44). It was worth to note only one study [27] conducted in the United States showed that early treatment with IVIG within 4 days was a protective factor against the developing CAL (RR, 0.67; 95% CI, 0.52–0.86; P = .002).

For the subgroup of the type of CALs (coronary artery dilation or CAA), eight non-randomized studies [9–11, 25, 29, 32–34] and two randomized trials [35, 36] with a sample size of 4122 participants, reported the outcomes of CAA in the acute phase (34 of 608 vs. 312 of 3514). Eight non-randomized studies [9, 11, 20, 22, 25, 29, 33, 34] with a sample size of 4235 participants reported the outcomes of CAA during 1–2 months follow-up (71 of 1625 vs. 126 of 2610). Both in the acute phase (RR, 0.23; 95% CI, 0.14–0.39; P < .00001; I^2^ = 65%) and during 1–2 months follow-up (RR, 0.20; 95% CI, 0.13–0.31; P < .00001; I^2^ = 43%), the pooled results showed that a significantly increased incidence of CAA was observed in the late IVIG (≥ 10 days) treatment compared with the earlier treatment (<10 days). Furthermore, the incidence of CAA was lower in the early group than in the late group but without statistical significance (acute phase: RR, 0.42; 95% CI, 0.16–1.11; P = .08; I^2^ = 90%; follow-up: RR, 0.28; 95% CI, 0.07–1.14; P = .08; I^2^ = 75%) when using the 7-day as the cut-off point. At the 4-day cut-off point, the incidence of CAAs was similar between the two groups (acute phase: RR, 0.91; 95% CI, 0.35–2.35; P = .84; I^2^ = 70%, follow-up: RR, 1.18; 95% CI, 0.52–2.65; P = .69; I^2^ = 24%). The detailed information can be seen in ***Supplementary Material, Appendix S12*** and ***Supplementary Material, Appendix S13***.

Eight non-randomized studies [9–11, 25, 29, 30, 32, 33] and three randomized trials [35, 36, 38] with a sample size of 4146 participants reported the outcomes of coronary artery dilation in the acute phase (89 of 637 vs. 553 of 3509). Six non-randomized studies [9, 11, 20, 25, 29, 33] with a sample size of 2907 participants reported the outcomes of coronary artery dilation during 1–2 months follow-up (41 of 502 vs. 229 of 2405). Both in the acute phase (RR, 0.64; 95% CI, 0.53–0.77; P < .00001; I^2^ = 0%) and during 1–2 months follow-up (RR, 0.51; 95% CI, 0.34–0.79; P = .002; I^2^ = 15%), the pooled analysis showed that delaying IVIG treatment for more than 10 days had a higher risk of coronary artery dilation than the earlier treatment within 10 days. There was no evidence of heterogeneity within the subgroups. Meanwhile, the incidence of coronary artery dilation in patients who were treated with IVIG within 4 days (acute phase: RR, 0.86; 95% CI, 0.67–1.10; P = .23; I^2^ = 0%, follow-up: RR, 0.80; 95% CI, 0.55–1.15; P = .22; I^2^ = 0%) or 7 days (acute phase: RR, 0.64; 95% CI, 0.40–1.02; P = .06; I^2^ = 50%, follow-up: RR, 0.79; 95% CI, 0.37–1.70; P = .55; I^2^ = 66%) of symptom onset was lower than the late IVIG treatment but without statistical significance. The results can be seen in ***Supplementary Material, Appendix S14*** and ***S15***.

## Publication Bias

Funnel plots were generated for outcomes with 10 or more studies to evaluate publication bias (***Supplementary Material, Appendix S16*** and ***Supplementary Material, Appendix S17***). The Egger test did not demonstrate significant publication bias for initial IVIG resistance (4-day cut-off point: P = .36, 10-day cut-off point: P = .90) and CALs in the acute phase (4-day cut-off point: P = .25, 10-day cut-off point: P = .57).

## Discussion

This systematic review and meta-analysis of 27 studies, including 41,139 participants, comprehensively summarized the available published literature and focused on the optimal therapeutic window of IVIG treatment across a wide range of populations. There are several main findings of our study. First, very low-quality evidence showed that the early IVIG treatment (≤ 4 days) had a higher IVIG resistance rate than the late treatment (≥ 5 days). Further subgroup analysis with heterogeneity from observational studies showed similar results. However, the pooled results of the RCTs subgroup showed that early IVIG treatment within 4 days was not associated with a higher risk of initial IVIG resistance. In contrast to the studies performed in China and Canada that found no significant difference in IVIG resistance, studies conducted in Japan and South Korea showed an increased risk of IVIG resistance with early IVIG treatment within 4 days. Second, early IVIG treatment within 7 days was associated with a lower occurrence of CALs in the acute phase in the overall estimate. Third, for the secondary outcomes, there was a lower risk of CALs during 1–2 months follow-up for those who started IVIG administration within 10 days from the onset.

IVIG is the first-line treatment of KD with well-established therapeutic effects in preventing coronary artery abnormalities [41]. However, the criteria for when to provide IVIG are unclear and differ from the latest guidelines. The latest 2017 American Heart Association (AHA) and 2018 Italian Society of Pediatrics guidelines recommend that IVIG be administered to KD patients within the first 10 days of illness and, if possible, within the first seven days of illness because the rate at which KD patients develop aneurysms increases significantly after the ninth day of illness [1, 7]. Similarly, the 2020 Japanese Circulation Society stated that IVIG was most frequently administered on the 5th day of illness [8]. There is no suggestion on the optimal timing of IVIG and if it can be given earlier. Our study showed that IVIG treatment within 4 days of illness was associated with increased initial IVIG resistance, which is in line with the results of a previous meta-analysis [42]. It is worth noting that subgroup analyses for the RCTs revealed that early IVIG treatment (≤ 4 days) was not associated with a higher risk of initial IVIG resistance. Meanwhile, the pooled results from observational studies with heterogeneity showed that early IVIG therapy (≤ 4 days ) was associated with a higher risk of IVIG resistance. Thus, these results must be interpreted cautiously since most of the observational studies were retrospective, with potential selection and information bias. More large, well-conducted RCT evaluating the relationship between the early IVIG treatment and IVIG resistance would answer this question.

In the nationwide surveys of KD in Japan, patients treated early (≤ day 4 of illness) were more likely to require retreatment with IVIG [12]. Several studies conducted in Japan showed similar results [13, 21]. On the other hand, recent studies [9, 10] showed that Chinese KD patients who had earlier IVIG treatment administration within 4 days might not increase the higher incidence of IVIG resistance. Further subgroup analyses confirmed that unlike the studies performed in China that found no significant difference in IVIG resistance, studies conducted in Japan showed an increased risk in the rate of IVIG resistance with early IVIG treatment within 4 days. One possible explanation is that the early group showed a higher rate of IVIG resistance because there might be more patients with severe inflammation or atypical clinical course, respectively. Another reason for the difference may be that KD’s characteristics vary between ethnicities.

The most common sequela of KD is CAL, which is speculated to be caused by acute systemic inflammation [43]. The early prevention of CALs is important to improve outcomes in KD patients, as CALs severely impair the life quality of KD patients. It is not surprising that our results showed that patients treated earlier (≤ 7 days) had a lower rate of occurrence of CALs in the acute phase. Furthermore, KD patients who received IVIG therapy more than 10 days after the onset had a higher incidence of CALs during 1–2 months of follow-up. Our study highlights the importance of early intervention to prevent coronary artery complications in treating KD. To minimize cardiac sequelae, avoiding any delay in IVIG treatment is crucial because earlier inflammatory suppression may contribute to avoiding developing CAA [44].

## Strengths and Limitations

Our study had strengths: First, we used standard Cochrane protocols and had the largest cumulative sample size to date compared to the previous reports. Second, we analyzed long-term follow-up data on primary and secondary outcomes and included all IVIG infusion time points. Third, no language limit was applied; we included studies published in English and Chinese. Fourth, the GRADEpro approach was used to rate the certainty of evidence.

This study had several limitations to be noted. First, Most of the articles we included were retrospective observational studies with potential selection and information bias. In addition, the sample size of included RCTs was small, and all were carried out in China. Second, the clinical heterogeneity of studies, particularly the participant characteristics, IVIG treatment protocol, and the diagnostic criteria of IVIG resistance and CALs, was quite varied, potentially leading to substantial heterogeneity. Finally, the certainty of the evidence for all outcomes was very low.

## Conclusion

The findings of this systematic review and meta-analysis pooling data from multiple countries demonstrate that IVIG therapy within 7 days of illness may be the optimal therapeutic window of IVIG. IVIG treatment within 7 days is found to be effective for reducing the risk of coronary artery lesions and cardiac sequelae in KD patients. The early IVIG treatment within 4 days should be vigilant for the IVIG resistance although large multi-center randomized trials with well design are needed.

## Electronic supplementary material

Below is the link to the electronic supplementary material.

Supplementary appendix file: Supplementary Material, Appendix S1. Search strategies. Supplementary Material, Appendix S2. List of references with final exclusion reasons. Supplementary Material, Appendix S3. IVIG treatment protocol and definitions of IVIG resistance. Supplementary Material, Appendix S4. Newcastle-Ottawa scale to rate risk of bias for cohort and case-control study. Supplementary Material, Appendix S5. Cochrane Risk of Bias tool 2.0 (RoB 2) to rate risk of bias for randomised trials. Supplementary Material, Appendix S6. Evidence profiles. Supplementary Material, Appendix S7. Forest Plot of risk ratios (RR) for CALs during 1–2 months follow-up for Early IVIG VS Late IVIG. Supplementary Material, Appendix S8. Sensitivity analysis of initial IVIG resistance. Supplementary Material, Appendix S9. Sensitivity analysis of CALs in acute phase. Supplementary Material, Appendix S10. Subgroup analysis of initial IVIG resistance. Supplementary Material, Appendix S11. Subgroup analysis of CALs in acute phase. Supplementary Material, Appendix S12. Subgroup analysis of CAA in the acute phase for Early IVIG VS Late IVIG. Supplementary Material, Appendix S13. Subgroup analysis of CAA during 1–2 months follow-up for Early IVIG VS Late IVIG. Supplementary Material, Appendix S14. Subgroup analysis of coronary artery dilation in acute phase for Early IVIG VS Late IVIG. Supplementary Material, Appendix S15. Subgroup analysis of coronary artery dilation during 1–2 months follow-up for Early IVIG VS Late IVIG. Supplementary Material, Appendix S16. Funnel plot of initial IVIG resistance. Supplementary Material, Appendix S17. Funnel plot of CALs in acute phase.

## Data Availability

The datasets used and/or analyzed during the current study are available within the manuscript.

## Abbreviations

KD: Kawasaki disease
IVIG: Intravenous immunoglobulin
CALs: Coronary artery lesions
CAA: Coronary artery aneurysm
RCTs: Randomized controlled trials
NOS: Newcastle-Ottawa scale
RoB: Risk of Bias Tool
AHA: American Heart Association
