# Supplementary material for: The therapeutic window of intravenous immunoglobulin (IVIG) and its correlation with clinical outcomes in Kawasaki disease: a systematic review and meta-analysis
Source: Ital J Pediatr. 2023 Apr 11;49:45. doi: 10.1186/s13052-023-01451-6 (PMC10088291; doi:10.1186/s13052-023-01451-6)
Supplement: Supplementary file 1 — Supplementary appendix file: Supplementary Material, Appendix S1. Search strategies. Supplementary Material, Appendix S2. List of references with final exclusion reasons. Supplementary Material, Appendix S3. IVIG treatment protocol and definitions of IVIG resistance. Supplementary Material, Appendix S4. Newcastle-Ottawa scale to rate risk of bias for cohort and case-control study. Supplementary Material, Appendix S5. Cochrane Risk of Bias tool 2.0 (RoB 2) to rate risk of bias for randomised trials. Supplementary Material, Appendix S6. Evidence profiles. Supplementary Material, Appendix S7. Forest Plot of risk ratios (RR) for CALs during 1–2 months follow-up for Early IVIG VS Late IVIG. Supplementary Material, Appendix S8. Sensitivity analysis of initial IVIG resistance. Supplementary Material, Appendix S9. Sensitivity analysis of CALs in acute phase. Supplementary Material, Appendix S10. Subgroup analysis of initial IVIG resistance. Supplementary Material, Appendix S11. Subgroup analysis of CALs in acute phase. Supplementary Material, Appendix S12. Subgroup analysis of CAA in the acute phase for Early IVIG VS Late IVIG. Supplementary Material, Appendix S13. Subgroup analysis of CAA during 1–2 months follow-up for Early IVIG VS Late IVIG. Supplementary Material, Appendix S14. Subgroup analysis of coronary artery dilation in acute phase for Early IVIG VS Late IVIG. Supplementary Material, Appendix S15. Subgroup analysis of coronary artery dilation during 1–2 months follow-up for Early IVIG VS Late IVIG. Supplementary Material, Appendix S16. Funnel plot of initial IVIG resistance. Supplementary Material, Appendix S17. Funnel plot of CALs in acute phase. [file 13052_2023_1451_MOESM1_ESM.docx]

**Contents of Supplementary Material:**

**Supplementary Material, Appendix S1. Search strategies.**

**Supplementary Material, Appendix S2. List of references with final exclusion reasons.**

**Supplementary Material, Appendix S3. IVIG treatment protocol and definitions of IVIG resistance.**

**Supplementary Material, Appendix S4. Newcastle-Ottawa scale to rate risk of bias for cohort and case-control study.**

**Supplementary Material, Appendix S5. Cochrane Risk of Bias tool 2.0 (RoB 2) to rate risk of bias for randomised trials.**

**Supplementary Material, Appendix S6. Evidence profiles.**

**Supplementary Material, Appendix S7. Forest Plot of risk ratios (RR) for CALs during 1-2 months follow-up for Early IVIG VS Late IVIG.**

**Supplementary Material, Appendix S8. Sensitivity analysis of initial IVIG resistance.**

**Supplementary Material, Appendix S9. Sensitivity analysis of CALs in acute phase.**

**Supplementary Material, Appendix S10. Subgroup analysis of initial IVIG resistance.**

**Supplementary Material, Appendix S11. Subgroup analysis of CALs in acute phase.**

**Supplementary Material, Appendix S12. Subgroup analysis of CAA in the acute phase for Early IVIG VS Late IVIG.**

**Supplementary Material, Appendix S13. Subgroup analysis of CAA during 1-2 months follow-up for Early IVIG VS Late IVIG.**

**Supplementary Material, Appendix S14. Subgroup analysis of coronary artery dilation in acute phase for Early IVIG VS Late IVIG.**

**Supplementary Material, Appendix S15. Subgroup analysis of coronary artery dilation during 1-2 months follow-up for Early IVIG VS Late IVIG.**

**Supplementary Material, Appendix S16. Funnel plot of initial IVIG resistance.**

**Supplementary Material, Appendix S17. Funnel plot of CALs in acute phase.**

**Supplementary Material, Appendix S1: Search strategies.**

**PubMed**

**Search Query**

**#1** (Immunoglobulins, Intravenous[Mesh]) OR (Antibodies, Intravenous[Title/Abstract]) OR (Intravenous Antibodies[Title/Abstract]) OR (Immune Globulin, Intravenous[Title/Abstract]) OR (Intravenous Immune Globulin[Title/Abstract]) OR (Intravenous Immunoglobulins[Title/Abstract]) OR (Intravenous IG[Title/Abstract]) OR (IV Immunoglobulins[Title/Abstract]) OR (Immunoglobulins, IV[Title/Abstract]) OR (IVIG[Title/Abstract]) OR (IV Immunoglobulin[Title/Abstract]) OR (Immunoglobulin, IV[Title/Abstract]) OR (Intravenous Immunoglobulin[Title/Abstract]) OR (Immunoglobulin, Intravenous[Title/Abstract]) OR (Flebogamma DIF[Title/Abstract]) OR (Gamunex[Title/Abstract]) OR (Globulin-N[Title/Abstract]) OR (Globulin N[Title/Abstract]) OR (Intraglobin[Title/Abstract]) OR (Intraglobin F[Title/Abstract]) OR (Intravenous Immunoglobulins, Human[Title/Abstract]) OR (Human Intravenous Immunoglobulins[Title/Abstract]) OR (Immunoglobulins, Human Intravenous[Title/Abstract]) OR (Immunoglobulins, Intravenous, Human[Title/Abstract]) OR (Human Intravenous Immunoglobulin[Title/Abstract]) OR (Immunoglobulin, Human Intravenous[Title/Abstract]) OR (Intravenous Immunoglobulin, Human[Title/Abstract]) OR (Gammagard[Title/Abstract]) OR (Gamimune[Title/Abstract]) OR (Gamimmune[Title/Abstract]) OR (Modified Immune Globulin[Title/Abstract]) OR (Anti-Echovirus Antibody[Title/Abstract]) OR (Privigen[Title/Abstract]) OR (Sandoglobulin[Title/Abstract]) OR (Venoglobulin[Title/Abstract]) OR (Venoglobulin-I[Title/Abstract]) OR (Venoglobulin I[Title/Abstract]) OR (Iveegam[Title/Abstract]) OR (Alphaglobin[Title/Abstract]) OR (Endobulin[Title/Abstract]) OR (Gamimune N[Title/Abstract]) OR (Gamimmune N[Title/Abstract]) OR (Gammonativ[Title/Abstract]) OR (intravenous gamma globulin[Title/Abstract]) OR (IVGG[Title/Abstract]) OR (Immunoglobulins[Mesh]) OR (Globulins, Immune[Title/Abstract]) OR (Immunoglobulin[Title/Abstract]) OR (Immune Globulin[Title/Abstract]) OR (Globulin, Immune[Title/Abstract]) OR (Immune Globulins[Title/Abstract]) OR (gamma-Globulins[Mesh]) OR (gamma Globulins[Title/Abstract]) OR (gamma-Globulin[Title/Abstract]) OR (gamma Globulin[Title/Abstract])

**#2** (Mucocutaneous Lymph Node Syndrome[Mesh]) OR (Kawasaki Syndrome[Title/Abstract]) OR (Lymph Node Syndrome, Mucocutaneous[Title/Abstract]) OR (Kawasaki Disease[Title/Abstract]) OR (KD[Title/Abstract]) OR (MCLS[Title/Abstract])

**#3** ((Mucocutaneous Lymph Node Syndrome[Mesh]) OR (Kawasaki Syndrome[Title/Abstract]) OR (Lymph Node Syndrome, Mucocutaneous[Title/Abstract]) OR (Kawasaki Disease[Title/Abstract]) OR (KD[Title/Abstract]) OR (MCLS[Title/Abstract])) AND ((Immunoglobulins, Intravenous[Mesh]) OR (Antibodies, Intravenous[Title/Abstract]) OR (Intravenous Antibodies[Title/Abstract]) OR (Immune Globulin, Intravenous[Title/Abstract]) OR (Intravenous Immune Globulin[Title/Abstract]) OR (Intravenous Immunoglobulins[Title/Abstract]) OR (Intravenous IG[Title/Abstract]) OR (IV Immunoglobulins[Title/Abstract]) OR (Immunoglobulins, IV[Title/Abstract]) OR (IVIG[Title/Abstract]) OR (IV Immunoglobulin[Title/Abstract]) OR (Immunoglobulin, IV[Title/Abstract]) OR (Intravenous Immunoglobulin[Title/Abstract]) OR (Immunoglobulin, Intravenous[Title/Abstract]) OR (Flebogamma DIF[Title/Abstract]) OR (Gamunex[Title/Abstract]) OR (Globulin-N[Title/Abstract]) OR (Globulin N[Title/Abstract]) OR (Intraglobin[Title/Abstract]) OR (Intraglobin F[Title/Abstract]) OR (Intravenous Immunoglobulins, Human[Title/Abstract]) OR (Human Intravenous Immunoglobulins[Title/Abstract]) OR (Immunoglobulins, Human Intravenous[Title/Abstract]) OR (Immunoglobulins, Intravenous, Human[Title/Abstract]) OR (Human Intravenous Immunoglobulin[Title/Abstract]) OR (Immunoglobulin, Human Intravenous[Title/Abstract]) OR (Intravenous Immunoglobulin, Human[Title/Abstract]) OR (Gammagard[Title/Abstract]) OR (Gamimune[Title/Abstract]) OR (Gamimmune[Title/Abstract]) OR (Modified Immune Globulin[Title/Abstract]) OR (Anti-Echovirus Antibody[Title/Abstract]) OR (Privigen[Title/Abstract]) OR (Sandoglobulin[Title/Abstract]) OR (Venoglobulin[Title/Abstract]) OR (Venoglobulin-I[Title/Abstract]) OR (Venoglobulin I[Title/Abstract]) OR (Iveegam[Title/Abstract]) OR (Alphaglobin[Title/Abstract]) OR (Endobulin[Title/Abstract]) OR (Gamimune N[Title/Abstract]) OR (Gamimmune N[Title/Abstract]) OR (Gammonativ[Title/Abstract]) OR (intravenous gamma globulin[Title/Abstract]) OR (IVGG[Title/Abstract]) OR (Immunoglobulins[Mesh]) OR (Globulins, Immune[Title/Abstract]) OR (Immunoglobulin[Title/Abstract]) OR (Immune Globulin[Title/Abstract]) OR (Globulin, Immune[Title/Abstract]) OR (Immune Globulins[Title/Abstract]) OR (gamma-Globulins[Mesh]) OR (gamma Globulins[Title/Abstract]) OR (gamma-Globulin[Title/Abstract]) OR (gamma Globulin[Title/Abstract]))

**Cochrane Library**

**ID Search**

**#1** (Mucocutaneous Lymph Node Syndrome):ti,ab,kw OR (Kawasaki Syndrome):ti,ab,kw OR (Lymph Node Syndrome, Mucocutaneous):ti,ab,kw OR (Kawasaki Disease):ti,ab,kw OR (Kawasaki diseases):ti,ab,kw OR (KD):ti,ab,kw OR (MCLS):ti,ab,kw

**#2** (Immunoglobulins, Intravenous):ti,ab,kw OR (Antibodies, Intravenous):ti,ab,kw OR (Intravenous Antibodies):ti,ab,kw OR (Immune Globulin, Intravenous):ti,ab,kw OR (Intravenous Immune Globulin):ti,ab,kw OR (Intravenous Immunoglobulins):ti,ab,kw OR (Intravenous IG):ti,ab,kw OR (IV Immunoglobulins):ti,ab,kw OR (Immunoglobulins, IV):ti,ab,kw OR (IVIG):ti,ab,kw OR (IV Immunoglobulin):ti,ab,kw OR (Immunoglobulin, IV):ti,ab,kw OR (Intravenous Immunoglobulin):ti,ab,kw OR (Immunoglobulin, Intravenous):ti,ab,kw OR (Flebogamma DIF):ti,ab,kw OR (Gamunex):ti,ab,kw OR (Globulin-N):ti,ab,kw OR (Globulin N):ti,ab,kw OR (Intraglobin):ti,ab,kw OR (Intraglobin F):ti,ab,kw OR (Intravenous Immunoglobulins, Human):ti,ab,kw OR (Human Intravenous Immunoglobulins):ti,ab,kw OR (Immunoglobulins, Human Intravenous):ti,ab,kw OR (Immunoglobulins, Intravenous, Human):ti,ab,kw OR (Human Intravenous Immunoglobulin):ti,ab,kw OR (Immunoglobulin, Human Intravenous):ti,ab,kw OR (Intravenous Immunoglobulin, Human):ti,ab,kw OR (Gammagard):ti,ab,kw OR (Gamimune):ti,ab,kw OR (Gamimmune):ti,ab,kw OR (Modified Immune Globulin):ti,ab,kw OR (Anti-Echovirus Antibody):ti,ab,kw OR (Privigen):ti,ab,kw OR (Sandoglobulin):ti,ab,kw OR (Venoglobulin):ti,ab,kw OR (Venoglobulin-I):ti,ab,kw OR (Venoglobulin I):ti,ab,kw OR (Iveegam):ti,ab,kw OR (Alphaglobin):ti,ab,kw OR (Endobulin):ti,ab,kw OR (Gamimune N):ti,ab,kw OR (Gamimmune N):ti,ab,kw OR (Gammonativ):ti,ab,kw OR (intravenous gamma globulin):ti,ab,kw OR (IVGG):ti,ab,kw OR (Immunoglobulins):ti,ab,kw OR (Globulins, Immune):ti,ab,kw OR (Immunoglobulin):ti,ab,kw OR (Immune Globulin):ti,ab,kw OR (Globulin, Immune):ti,ab,kw OR (Immune Globulins):ti,ab,kw OR (gamma-Globulins):ti,ab,kw OR (gamma Globulins):ti,ab,kw OR (gamma-Globulin):ti,ab,kw OR (gamma Globulin):ti,ab,kw

**#3** #1 AND #2

**Web of Science**

**ID Search**

**#1** TS=(Mucocutaneous Lymph Node Syndrome OR Kawasaki Syndrome OR Lymph Node Syndrome, Mucocutaneous OR Kawasaki Disease OR Kawasaki diseases OR KD OR MCLS)

**#2** TS=(Immunoglobulins, Intravenous OR Antibodies, Intravenous OR Intravenous Antibodies OR Immune Globulin, Intravenous OR Intravenous Immune Globulin OR Intravenous Immunoglobulins OR Intravenous IG OR IV Immunoglobulins OR Immunoglobulins, IV OR IVIG OR IV Immunoglobulin OR Immunoglobulin, IV OR Intravenous Immunoglobulin OR Immunoglobulin, Intravenous OR Flebogamma DIF OR Gamunex OR Globulin-N OR Globulin N OR Intraglobin OR Intraglobin F OR Intravenous Immunoglobulins, Human OR Human Intravenous Immunoglobulins OR Immunoglobulins, Human Intravenous OR Immunoglobulins, Intravenous, Human OR Human Intravenous Immunoglobulin OR Immunoglobulin, Human Intravenous OR Intravenous Immunoglobulin, Human OR Gammagard OR Gamimune OR Gamimmune OR Modified Immune Globulin OR Anti-Echovirus Antibody OR Privigen OR Sandoglobulin OR Venoglobulin OR Venoglobulin-I OR Venoglobulin I OR Iveegam OR Alphaglobin OR Endobulin OR Gamimune N OR Gamimmune N OR Gammonativ OR intravenous gamma globulin OR IVGG OR Immunoglobulins OR Globulins, Immune OR Immunoglobulin OR Immune Globulin OR Globulin, Immune OR Immune Globulins OR gamma-Globulins OR gamma Globulins OR gamma-Globulin OR gamma Globulin)

**#3** #2 AND #1

**Embase**

**No.** **Query**

#1 'immunoglobulins, intravenous':ab,ti OR 'antibodies, intravenous':ab,ti OR 'intravenous antibodies':ab,ti OR 'immune globulin, intravenous':ab,ti OR 'intravenous immune globulin':ab,ti OR 'intravenous immunoglobulins':ab,ti OR 'intravenous ig':ab,ti OR 'iv immunoglobulins':ab,ti OR 'immunoglobulins, iv':ab,ti OR 'ivig':ab,ti OR 'iv immunoglobulin':ab,ti OR 'immunoglobulin, iv':ab,ti OR 'intravenous immunoglobulin':ab,ti OR 'immunoglobulin, intravenous':ab,ti OR 'flebogamma dif':ab,ti OR 'gamunex':ab,ti OR 'globulin-n':ab,ti OR 'globulin n':ab,ti OR 'intraglobin':ab,ti OR 'intraglobin f':ab,ti OR 'intravenous immunoglobulins, human':ab,ti OR 'human intravenous immunoglobulins':ab,ti OR 'immunoglobulins, human intravenous':ab,ti OR 'immunoglobulins, intravenous, human':ab,ti OR 'human intravenous immunoglobulin':ab,ti OR 'immunoglobulin, human intravenous':ab,ti OR 'intravenous immunoglobulin, human':ab,ti OR 'gammagard':ab,ti OR 'gamimune':ab,ti OR 'gamimmune':ab,ti OR 'modified immune globulin':ab,ti OR 'anti-echovirus antibody':ab,ti OR 'privigen':ab,ti OR 'sandoglobulin':ab,ti OR 'venoglobulin':ab,ti OR 'venoglobulin-i':ab,ti OR 'venoglobulin i':ab,ti OR 'iveegam':ab,ti OR 'alphaglobin':ab,ti OR 'endobulin':ab,ti OR 'gamimune n':ab,ti OR 'gamimmune n':ab,ti OR 'gammonativ':ab,ti OR 'intravenous gamma globulin':ab,ti OR 'ivgg':ab,ti OR 'immunoglobulins':ab,ti OR 'globulins, immune':ab,ti OR 'immunoglobulin':ab,ti OR 'immune globulin':ab,ti OR 'globulin, immune':ab,ti OR 'immune globulins':ab,ti OR 'gamma-globulins':ab,ti OR 'gamma globulins':ab,ti OR 'gamma-globulin':ab,ti OR 'gamma globulin':ab,ti

#2 'mucocutaneous lymph node syndrome':ab,ti OR 'kawasaki syndrome':ab,ti OR 'lymph node syndrome, mucocutaneous':ab,ti OR 'kawasaki disease':ab,ti OR 'kawasaki diseases':ab,ti OR 'kd':ab,ti OR 'mcls':ab,ti

#3 #1 AND #2

**Chinese Biomedical Literature database (CBM) - field searching in Chinese**

**序号 检索表达式**

1) "黏膜皮肤淋巴结综合征"[不加权:扩展] OR "川崎病"[常用字段:智能] OR "淋巴结综合征"[常用字段:智能] OR "黏膜皮肤"[常用字段:智能] OR "KD"[常用字段:智能] OR "MCLS"[常用字段:智能]

2) "免疫球蛋白"[常用字段:智能] OR "丙种球蛋白"[常用字段:智能] OR "γ球蛋白"[常用字段:智能] OR "IVIG"[常用字段:智能] OR "IVGG"[常用字段:智能]

3) ("免疫球蛋白"[常用字段:智能] OR "丙种球蛋白"[常用字段:智能] OR "γ球蛋白"[常用字段:智能] OR "IVIG"[常用字段:智能] OR "IVGG"[常用字段:智能]) AND ("黏膜皮肤淋巴结综合征"[不加权:扩展] OR "川崎病"[常用字段:智能] OR "淋巴结综合征"[常用字段:智能] OR "黏膜皮肤"[常用字段:智能] OR "KD"[常用字段:智能] OR "MCLS"[常用字段:智能])

4) (("免疫球蛋白"[常用字段:智能] OR "丙种球蛋白"[常用字段:智能] OR "γ球蛋白"[常用字段:智能] OR "IVIG"[常用字段:智能] OR "IVGG"[常用字段:智能]) AND ("黏膜皮肤淋巴结综合征"[不加权:扩展] OR "川崎病"[常用字段:智能] OR "淋巴结综合征"[常用字段:智能] OR "黏膜皮肤"[常用字段:智能] OR "KD"[常用字段:智能] OR "MCLS"[常用字段:智能])) AND ("临床试验"[文献类型] OR "随机对照试验"[文献类型] OR "多中心研究"[文献类型])

**Supplementary Material, Appendix S2: List of references with final exclusion reasons.**

| **No.** | **Studies** | **First author** | **Reasons of exclusion** | **Publication year** |
| --- | --- | --- | --- | --- |
| 01 | Association between duration of fever before treatment and intravenous immunoglobulin resistance in Kawasaki disease. | Wang | Comparison between drug-resistance group and non-drug-resistance group | 2022 |
| 02 | Factors affecting the duration of coronary artery lesions in patients with the Kawasaki disease: a retrospective cohort study. | Zhang | Comparison between dilation group and aneurysm group | 2021 |
| 03 | Effect of early intravenous immunoglobulin in the treatment of Kawasaki disease and analysis of the effects on immune function and inflammatory state in children. | Yu | Lack of outcomes | 2021 |
| 04 | Effect of early intravenous immunoglobulin on Kawasaki disease and the influence of serum inflammatory index and immune index in children. | Liao | Lack of outcomes | 2020 |
| 05 | Immunoglobulin for Kawasaki disease: A 3-year retrospective audit. | Pascall | Lack of control group | 2019 |
| 06 | Effect of different application time of gamma globulin on therapeutic effect of Kawasaki disease in children. | Hu | Lack of outcomes | 2019 |
| 07 | Delayed intravenous immunoglobulin treatment increased the risk of coronary artery lesions in children with Kawasaki disease at different status. | Qiu | Lack of outcomes | 2018 |
| 08 | Cardiac Complications, Earlier Treatment, and Initial Disease Severity  in Kawasaki Disease. | Abrams | Not available for the baseline data and outcomes | 2017 |
| 09 | Early Immunoglobulin Therapy and Outcomes in Kawasaki Disease. | Ho | Lack of outcomes | 2015 |
| 10 | Clinical study of gamma globulin in treatment of Kawasaki disease. | Zhang | Lack of control group | 2010 |
| 11 | Early intravenous gamma globulin retreatment for refractory Kawasaki disease. | Chiyonobu | Comparison between resistant group and responsive group | 2003 |
| 12 | Association between duration of fever before treatment and intravenous immunoglobulin resistance in Kawasaki disease Chinese Journal of Contemporary Pediatrics. | Hu | Comparison between conventional treatment group and conventional treatment+ IVIG group | 2002 |
| 13 | Use of intravenous gamma-globulin for Kawasaki disease: effects on cardiac sequelae. | Yanagawa | Lack of control group | 1997 |
| 14 | Optimal dosage and differences in therapeutic efficacy of IGlV in  Kawasaki disease. | Onouchi | Optimal dose of IGIV; Chemically  modified IVIG VS native IGIV | 1995 |
| 15 | Study on coronary artery lesions in patients with Kawasaki disease recent 9 years experience. | Wu | Aspirin VS aspirin+different doses of IVIG | 1993 |
| 16 | Intravenous gamma-globulin treatment in Kawasaki disease. | Harada | Study design according to study stages and doses of γ-globulin | 1991 |
| 17 | The treatment of Kawasaki syndrome with intravenous gamma globulin. | Newburger | Comparison between aspirin group and gamma globulin group | 1986 |
| 18 | High-dose intravenous gammaglobulin for Kawasaki disease. | Furusho | Comparison between aspirin group and IVIG group | 1984 |

**Supplementary Material, Appendix S3:** **IVIG treatment protocol and definitions of IVIG resistance.**

| **Author** | **Year** | **Treatment protocol** | **Definitions of IVIG resistance** |
| --- | --- | --- | --- |
| Cai | 2022 | All KD patients received the standard therapy with IVIG (2 g/kg) and aspirin (30-50 mg/kg/d during the acute phase of illness) immediately after the diagnosis. The aspirin was lowered to 3-5 mg/kg/d 2-3 days after the patients were afebrile. Other therapies, including prednisolone, were not used in the initial treatment. Combined antiplatelet and anticoagulation therapy were recommended for patients with giant aneurysms. For IVIG-resistant patients, the 2nd IVIG of the same dosage was administrated. If fever persists 36h after the 2nd IVIG infusion, intravenous methylprednisolone (30 mg/kg/dose) was performed for 3 consecutive days. No patients received additional treatment such as infliximab, plasma exchange, and cytotoxic agents. | IVIG resistance was defined as recrudescent or persistent fever ≥36 hours but not longer than 7 days after initial IVIG infusion. |
| Li | 2021 | The treatment protocol included high doses of IVIG (2 g/kg) as a single infusion over 8 to 12 h along with aspirin (30–50 mg/kg per day) during the acute phase followed by 3–5 mg/kg per day for 6–8 weeks or until resolution of coronary artery abnormalities. Combined antiplatelet and anticoagulation therapy were recommended for patients with giant aneurysms. | Patients with KD develop a recrudescent or persistent fever at least 36 hours after the end of their IVIG infusion and are termed as IVIG-resistant KD. |
| Yazdi | 2021 | In the acute phase, a combination of intravenous immunoglobulin (Green Cross, 2 g/kg, 12 hours) and oral ASA (80-100 mg/kg/day, three times a day)  (Osvah Co, Tehran, Iran) were administered. When a febrile condition continued for 48 hours, low-dose ASA (3–5 mg/kg/day, once daily) was replaced with the previous high-dose regimen for eight weeks. It should be noted that all of the patients were treated with IVIG and ASA immediately after a diagnosis of KD was confirmed. | Not mention |
| Ha | 2020 | Patients fulfilled the diagnostic criteria for KD were treated with IVIG (a single 2 g/kg dose). | Patients who remained febrile for >48 h after receiving IVIG were defined as IVIG non-responders. |
| Shiozawa | 2018 | All the patients were first treated with 2 g/kg IVIG. Flurbiprofen and dipyridamole were sometimes used as an alternative to aspirin in patients with liver damage. Other therapies including prednisolone were not used in the initial treatment. No patient required four or more doses of IVIG. | IVIG resistance was defined as the need for additional treatment because of persistent fever or relapsing fever associated with other KD symptoms after resolution of fever. |
| Kuwabara | 2018 | Not mention | Not mention |
| Downie | 2017 | Treated patients received standard first line therapy for KD, which consisted of 2 g/kg IVIG and high dose (80–100 mg/kg) acetylsalicylic acid (ASA), though some patients received this treatment after >10 days of fever. | Non-response to treatment was defined as axillary temperature ≥ 37.5 °C for >24 h after the end of the initial IVIG infusion. |
| Mohammadzadeh | 2016 | The treatment used for all patients was the standard treatment of single-dose IVIg (2 g/kg) over 10-12 hours and aspirin (100 mg/kg/day in four divided doses) for two weeks and then 5 mg/kg/day for two months. Since the fever continued in nine patients (9%), they received the second dose of IVIG. Moreover, among these nine patients, fever continued in one patient despite receiving two doses of IVIg, and thus, the pulse therapy of methyl prednisolone (30mg/Kg/day) was applied. | Not mention |
| Chen | 2015 | The regimens of 2000mg/kg once in 1148 cases (52.8%), 1000 mg/kg twice in 875 cases (40.3%), 1000 mg/kg once in 63 cases (2.9%), 400mg/kg/d or 500 mg/kg/d for 5 consecutive days in 3 cases (0.1%) and irregular usage in 84 cases (3.9%). | Not mention |
| Bal | 2014 | All children with confirmed KD received 2 g/kg of IVIG (Carimune, Flebogamma or Gamunex) over 12 hours. Aspirin (100 mg/kg) was given orally until the patients became afebrile for 96 hours followed by 4-6 mg per kg of body weight until 6 weeks after onset of illness. Children with abnormal ECHO continuously received aspirin (4-6 mg/kg) until resolution of coronary abnormality on echocardiogram. | We collected evidence of non-therapeutic responses characterized by persistence or recrudescence of fever lasting >36 hours after completion of the IVIG infusion. |
| Wang | 2014 | The patients received IVIG (2 g/kg) as a single infusion (10-12 hous) within 5 to 9 days of illness; if fever persists 48 hours after the 1nd IVIG, they received the second dose of IVIG. Observation group received 200 mg/kg of IVIG (Qilu Pharmaceutical No.S20092012, 100mg/pcs), as a single infusion (10-12 hous) within 24 hours and aspirin (30 mg/kg/d) was given orally (North China Pharmaceutical, 2010-2-34, 25 mg/pc). The control group received IVIG more than 5 days, and the others were the same as the observation group. | IVIG resistance was defined as recrudescent within 2 to 7 days, or persistent fever ≥ 38°C for ＞48 h after the end of IVIG infusion. |
| Callinan | 2012 | Not mention | Not mention |
| Sittiwangkul | 2011 | Treatment included a single dose of IVIG at 2 g/kg/dose within 12 hours and acetyl salicylic acid at 70–90 mg/kg/day. An additional dose of 2 g/kg of IVIG or steroid treatment was instituted in resistant KD when appropriate. | An additional dose of 2 g/kg of IVIG or steroid treatment was instituted in resistant KD when appropriate. |
| Du | 2009 | At the acute stage, patients were treated with IVIG on the basis of oral aspirin 30-50 mg/kg/d. | Non-response to treatment was defined as axillary temperature ≥ 38.5°C for > 48 h after the end of the initial IVIG infusion. |
| Muta | 2004 | The following regimens were frequently used in this study: 200 mg/kg for 5 days (7% of patients), 400 mg/kg for 5 days (51%), 1000 mg/kg for 1 day(11%), 1000 mg/kg for 2 days (14%), and 2000 mg/kg for 1day (12%). | Additional IVGG treatment |
| Li | 2006 | All KD patients received the therapy with IVIG (1 g/kg) and aspirin (50-100 mg/kg/d); if fever persists 48 hous after the 1nd IVIG, they received the second dose of IVIG (1 g/kg). | Re-treatments of IVGG. |
| Hsieh | 2004 | All patients were treated with high dose IVIG (2 g/kg) as a single infusion for 10 to 12 hours without concomitant aspirin therapy. After the fever subsided, low dose aspirin (3-5 mg/kg per day) was prescribed. | Patients whose fever persisted for 3 days after IVIG treatment were defined as the IVIG non-responders. |
| Nomura | 2002 | Basic IVGG treatment included the administration of 200 mg/kg per day for 5 days between 1989 and 1994 and 400 mg/kg per day between 1995 and 1998. Gamma globulin therapy was continued for at least 3 days and was stopped when the fever disappeared (body temperature under 37.5°C for at least 24 h). | Re-treatments of IVGG. |
| Muta | 2012 | Eighty-one percent of patients were treated with 2 g/kg. There were no patients who received adjunctive treatments with the initial IVIG other than aspirin. | Additional IVGG treatments. |
| Fong | 2004 | All patients with Kawasaki disease admitted to this hospital were treated with a standard dose of immunoglobulin (2 g/kg) and high dose aspirin (80–100 mg/kg/day) until the fever subsided for 48 hours and then were treated with low-dose aspirin. Retreatment was a dose of 2 g/kg and usually supported by persistently raised neutrophils and C-reactive protein. | Immunoglobulin retreatment was given if fever (> 38.5℃) and other signs persisted for 48 hours after the completion of the first dose of immunoglobulin or recrudescent fever. |
| Tse | 2002 | Initial treatment consisted of IVIG (2 g/kg) and oral ASA (100 mg/kg per day in 4 divided doses). Upon defervescence for 24 hours, the dosage of oral ASA was reduced to 3 to 5 mg/kg per day for 6 weeks. | Patients failing to defervesce within 24 hours after the completion of the IVIG infusion or those with KD recrudescence were treated with repeat IVIG and/or intravenous corticosteroids. |
| Kong | 2021 | All KD patients received the routine therapy with aspirin and glucocorticoid hormone, etc. Besides, observation group within 5 to 7 days of illness and control group within 8 to 10 days of illness received IVIG (2 g/kg), the initial drip rate was 2 ml/(kg·h), over 15 minutes, if there were no adverse reactions, adjust the drip rate as 4 ml/(kg·h), IVIG treatment time was within 10 hours. | Non-response to treatment was defined as temperature > 38.5 °C for > 48 h after the end of the initial IVIG infusion. |
| Wu | 2019 | All KD patients received the therapy with IVIG (2 g/kg) as a single infusion (observation group within 1 to 4 days of illness, control groupI within 5 to 9 days of illness, control groupII over 10 days of illness) immediately after the diagnosis, and oral aspirin [30-50 mg/(kg·d) in 4 divided doses] during the acute phase of illness. The aspirin was lowered to 3-5 mg/(kg·d) after the fever disappeared. | Non-response to treatment was defined as temperature ≥ 38.5°C for > 48 h after the end of the initial IVIG infusion. |
| Xiong | 2019 | All KD patients (observation group within 5 to 10 days of illness, control group over 10 days of illness) received the therapy with 1 g/(kg·d) IVIG (Lanzhou Institute of Biological, National Medicine Permit No.S20023015) as a single infusion in 8-12 hours. One week makes a course, two weeks in total. | Not mention |
| Shao | 2018 | Two groups received the routine therapy with aspirin and glucocorticoid hormone, etc. Furthermore, control group within 10 to 14 days of illness and observation group within 5 to 9 days of illness received IVIG (2 g/kg, 20-30 ml/min) over 10 minutes, if there were no adverse reactions, adjust the drip rate, IVIG was infused intravenously for 8-12 hours. The two groups use the same treament methods. | Not mention |
| An | 2017 | All patients with Kawasaki disease were treated with maintain water-electrolyte, acid-base balance, etc. Anti-infective treatment was given to the infected patients. Oral aspirin [30-50 mg/(kg·d) in 2-3 divided doses] was taken as basic adjunctive treatments immediately after admission, gradually reducing the dosage depending on the condition after the inflammatory response was controlled and defervescence for 3 days. After 3 weeks, it was reduced to 3-5 mg/(kg·d), until laboratory test parameters like erythrocyte sedimentation rate and platelet count returned to normal, the treatment lasted for 6-8 weeks. Combination a single dose of intravenous immunoglobulin (1 g/(kg·d), 8-12 hours) for 2 days. | Not mention |
| Li | 2013 | At the acute stage, patients were treated with oral aspirin 80-100 mg/kg per day, at the same time received IVIG (1 g/kg per day, 0.8-1.4 ml/min, 8-10 hours). | Non-response to treatment was defined as temperature ≥ 38.5°C for > 48 h after the end of the initial IVIG infusion. |

**Supplementary Material, Appendix S4: Newcastle-Ottawa scale to rate risk of bias for cohort and case-control study.**

| **Study** | **Selection** | **Comparability** | **Exposure/Outcome** | **Overall Rating**  **(more stars = lower risk of bias)** |
| --- | --- | --- | --- | --- |
| Cai 2022 | ★★★★ | ★ | ★★★ | ★★★★★★★★ |
| Li 2021 | ★★★★ | -^*^ | ★★★ | ★★★★★★★ |
| Yazdi 2020 | ★★★ | - | ★★★ | ★★★★★★ |
| Ha 2020 | ★★★★ | - | ★★★ | ★★★★★★★ |
| Shiozawa 2018 | ★★★★ | ★★ | ★★★ | ★★★★★★★★★ |
| Kuwabara 2018 | ★★★★ | - | ★★★ | ★★★★★★★ |
| Downie 2017 | ★★★★ | ★ | ★★★ | ★★★★★★★★ |
| Mohammadzadeh 2016 | ★★★★ | - | ★★★ | ★★★★★★★ |
| Chen 2015 | ★★★★ | - | ★★★ | ★★★★★★★ |
| Bal 2014 | ★★★★ | ★ | ★★★ | ★★★★★★★★ |
| Wang 2014 | ★★★★ | - | ★★★ | ★★★★★★★ |
| Callinan 2012 | ★★★★ | - | ★★★ | ★★★★★★★ |
| Sittiwangkul 2011 | ★★★★ | ★ | ★★★ | ★★★★★★★★ |
| Du 2009 | ★★★★ | - | ★★★ | ★★★★★★★ |
| Muta 2004 | ★★★★ | - | ★★★ | ★★★★★★★ |
| Li 2006 | ★★★★ | ★ | ★★★ | ★★★★★★★★ |
| Hsieh 2004 | ★★★★ | ★ | ★★★ | ★★★★★★★★ |
| Nomura 2002 | ★★★★ | ★ | ★★★ | ★★★★★★★★ |
| Muta 2012 | ★★★★ | ★ | ★★★ | ★★★★★★★★ |
| Fong 2004 | ★★★ | ★ | ★★★ | ★★★★★★★ |
| Tse 2002 | ★★★★ | ★ | ★★★ | ★★★★★★★★ |

^*^A single dash (-) indicates no stars. More stars equalling lower risk.

**Supplementary Material, Appendix S5: Cochrane Risk of Bias tool 2.0 (RoB 2) to rate risk of bias for randomised trials.**


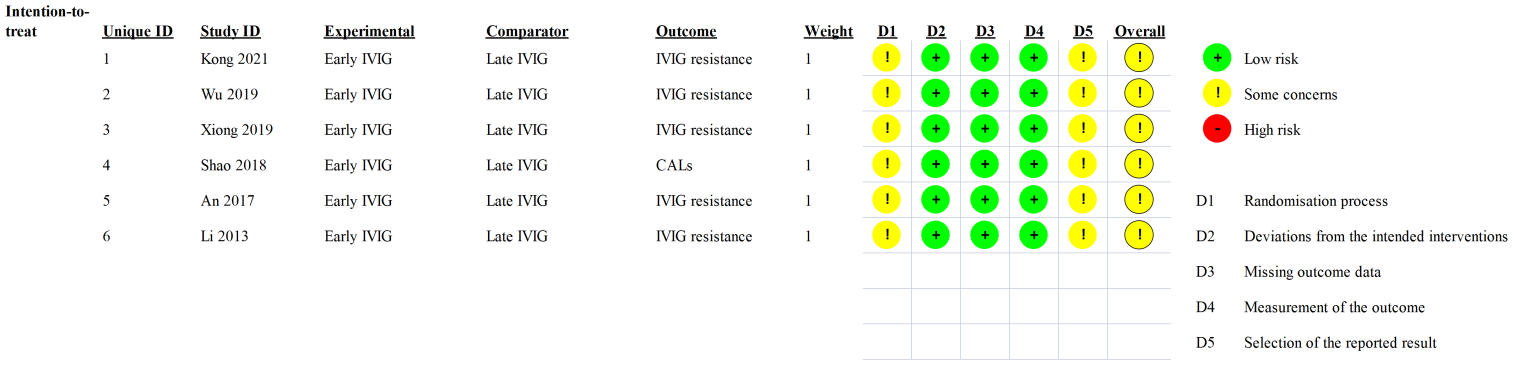

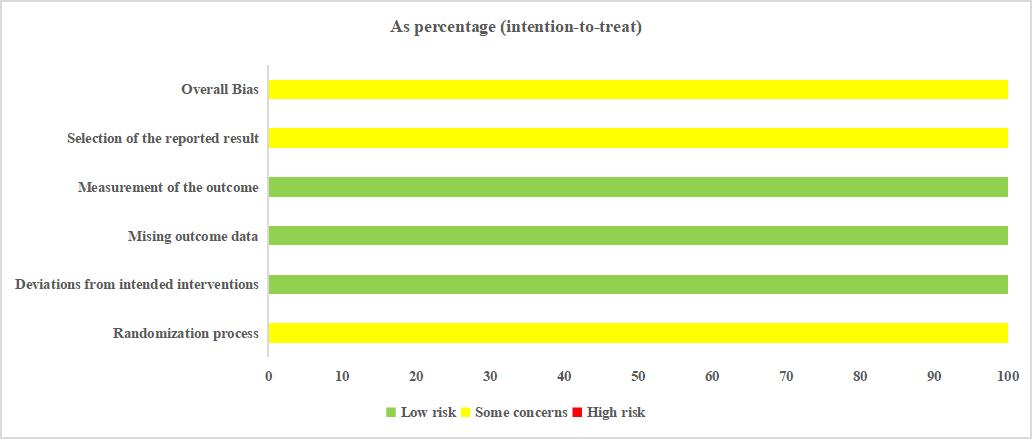


**Supplementary Material, Appendix S6: Evidence profiles.**

| **Certainty assessment** | | | | | | | **№ of patients** | | **Effect** | | **Certainty** | **Importance** |
| --- | --- | --- | --- | --- | --- | --- | --- | --- | --- | --- | --- | --- |
| **№ of studies** | **Study design** | **Risk of bias** | **Inconsistency** | **Indirectness** | **Imprecision** | **Other considerations** | **Early IVIG** | **Late IVIG** | **Relative**  **(95% CI)** | **Absolute**  **(95% CI)** |  |  |
| **Initial IVIG resistance (≤ 4 days VS ≥ 5 days)** | | | | | | | | | | | | |
| 3 | randomised trials | serious | very serious | not serious | extremely serious | none | 43/103 (41.7%) | 42/220 (19.1%) | **RR 1.97**  (0.76 to 5.07) | **185 more per 1,000**  (from 46 fewer to 777 more) | ⨁◯◯◯  Very low | CRITICAL |
| **CALs in acute phase (≤ 4 days VS ≥ 5days)** | | | | | | | | | | | | |
| 3 | randomised trials | serious | not serious | not serious | very serious | none | 11/103 (10.7%) | 32/220 (14.5%) | **RR 0.71**  (0.38 to 1.33) | **42 fewer per 1,000**  (from 90 fewer to 48 more) | ⨁◯◯◯  Very low | CRITICAL |

| **Certainty assessment** | | | | | | | **№ of patients** | | **Effect** | | **Certainty** | **Importance** |
| --- | --- | --- | --- | --- | --- | --- | --- | --- | --- | --- | --- | --- |
| **№ of studies** | **Study design** | **Risk of bias** | **Inconsistency** | **Indirectness** | **Imprecision** | **Other considerations** | **Early IVIG** | **Late IVIG** | **Relative**  **(95% CI)** | **Absolute**  **(95% CI)** |  |  |
| **Initial IVIG resistance ( ≤ 4 days VS ≥5 days)** | | | | | | | | | | | | |
| 13 | observational studies | not serious | serious | not serious | not serious | none | 2699/12481 (21.6%) | 2720/22277 (12.2%) | **RR 1.80**  (1.50 to 2.15) | **98 more per 1,000**  (from 61 more to 140 more) | ⨁◯◯◯  Very low | CRITICAL |
| **CALs in acute phase (≤ 4 days VS ≥ 5 days)** | | | | | | | | | | | | |
| 11 | observational studies | not serious | serious | not serious | serious | none | 1437/12936 (11.1%) | 2509/24640 (10.2%) | **RR 0.92**  (0.77 to 1.10) | **8 fewer per 1,000**  (from 23 fewer to 10 more) | ⨁◯◯◯  Very low | IMPORTANT |

**Supplementary Material, Appendix S7: Forest Plot of risk ratios (RR) for CALs during 1-2 months follow-up for Early IVIG VS Late IVIG.**

**
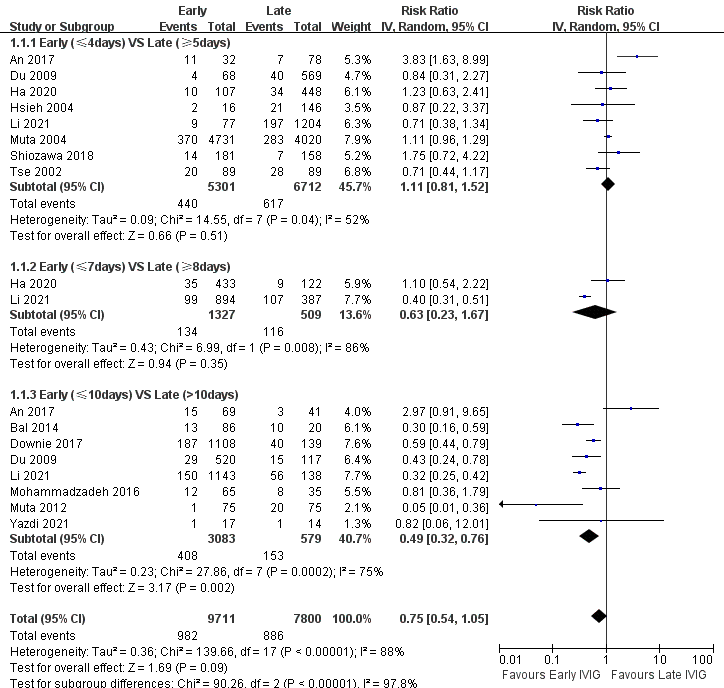
**

**Supplementary Material, Appendix S8: Sensitivity analysis of initial IVIG resistance.**

**Appendix S8-A Early IVIG (≤4days) VS Late IVIG (≥5days)**


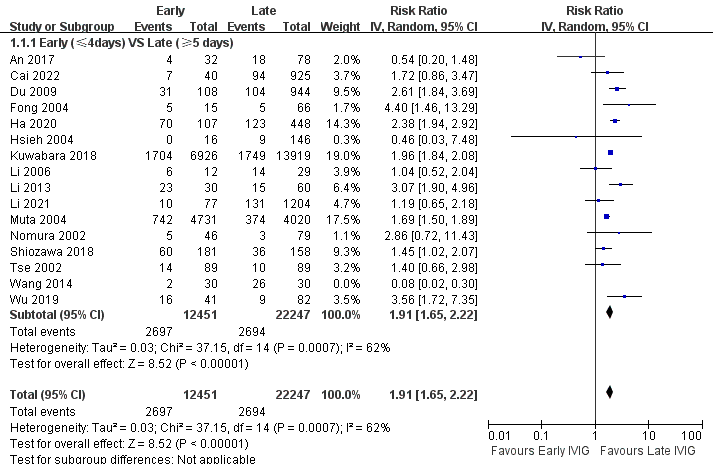


**Appendix S8-B Early IVIG (≤7days) VS Late IVIG (≥8days)**


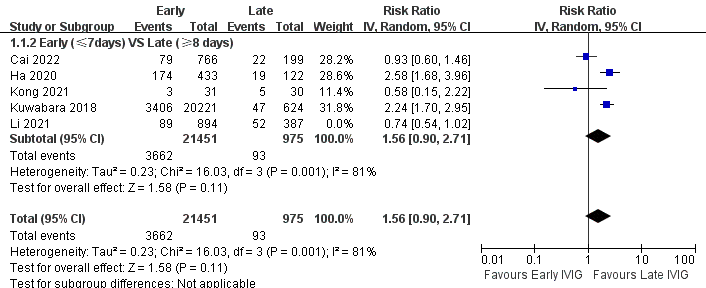


**Appendix S8-C Early IVIG (≤10days) VS Late IVIG (>10days)**


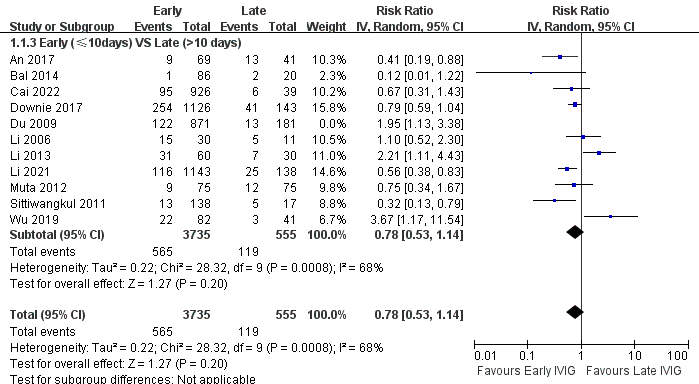


**Supplementary Material, Appendix S9: Sensitivity analysis of CALs in acute phase.**

**Appendix S9-A Early IVIG (≤4days) VS Late IVIG (≥5days)**


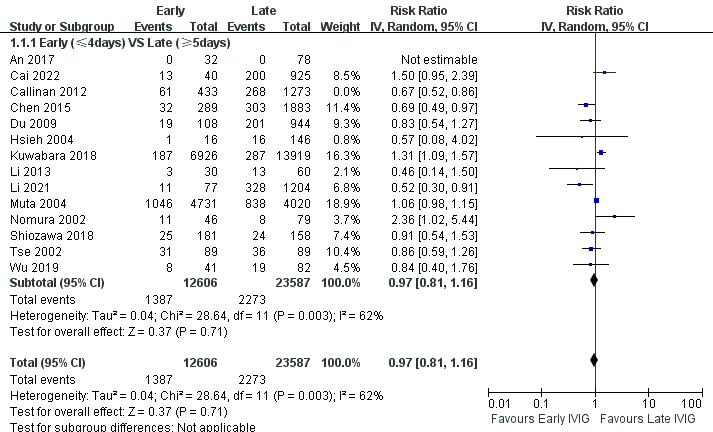


**Appendix S9-B Early IVIG (≤7days) VS Late IVIG (≥8days)**

**
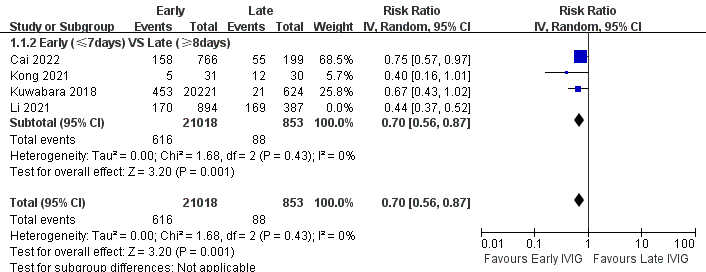
**

**Appendix S9-C Early IVIG (≤10days) VS Late IVIG (>10days)**

**
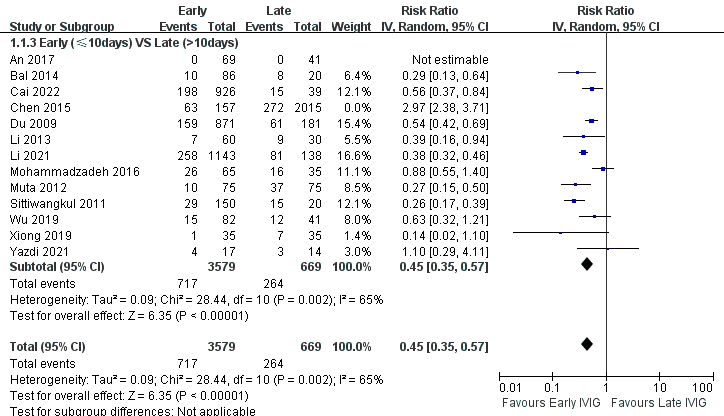
**

**Appendix S10: Subgroup analysis of initial IVIG resistance.**

**Appendix S10-A Subgroup analysis basing on research types**

**
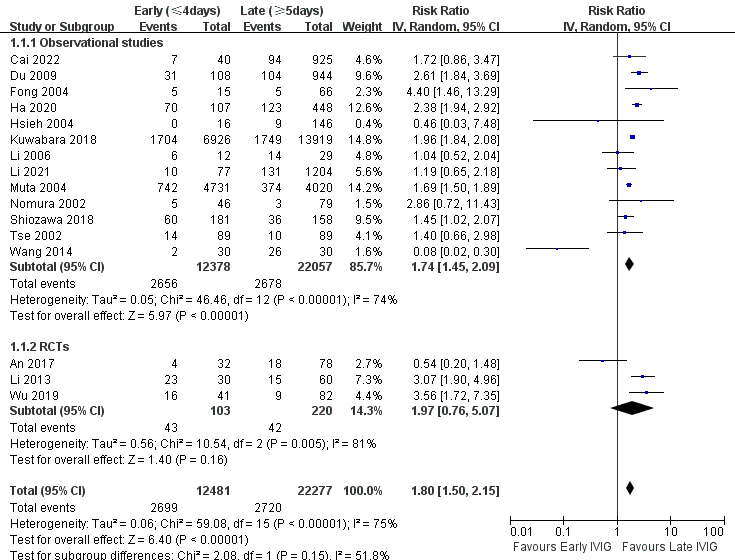
**

**Appendix S10-B Subgroup analysis basing on the study location**

**
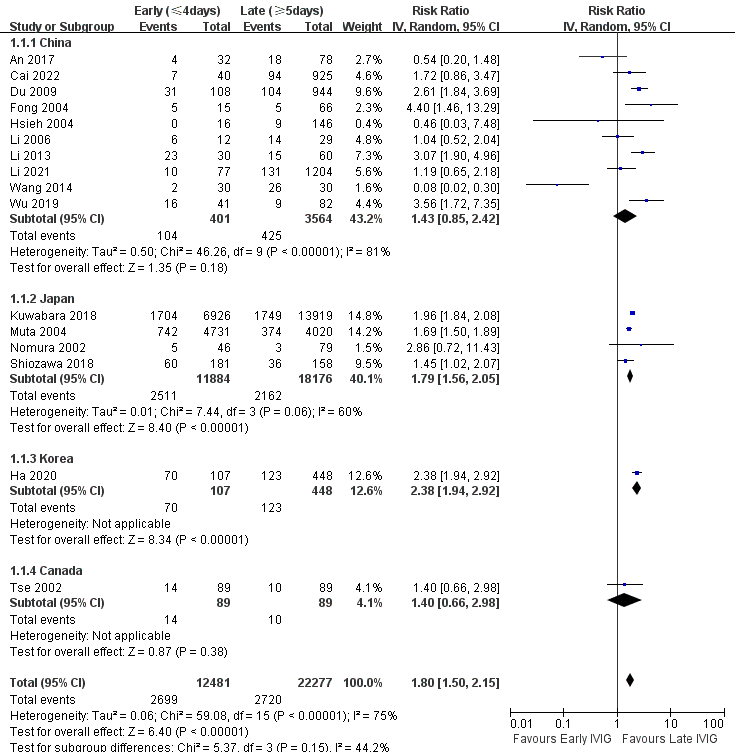
**

**Supplementary Material, Appendix S11: Subgroup analysis of CALs in acute phase.**

**Appendix S11-A Subgroup analysis basing on research types**

**
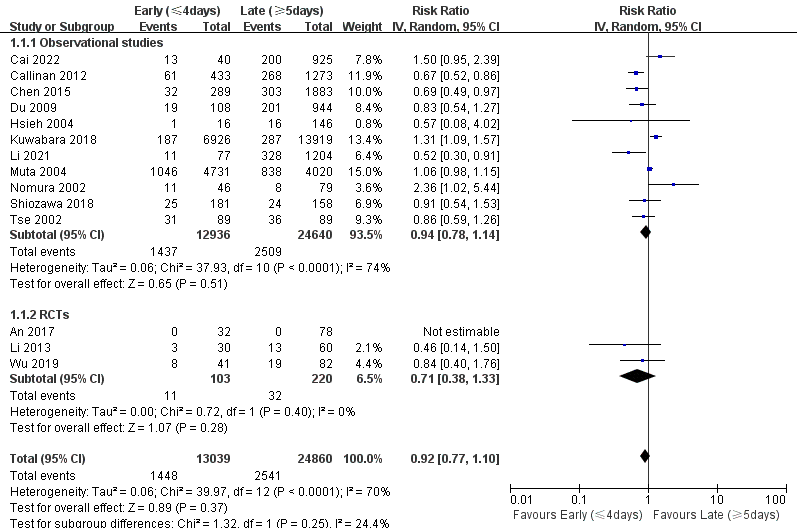
**

**Appendix S11-B Subgroup analysis basing on the study location**

**
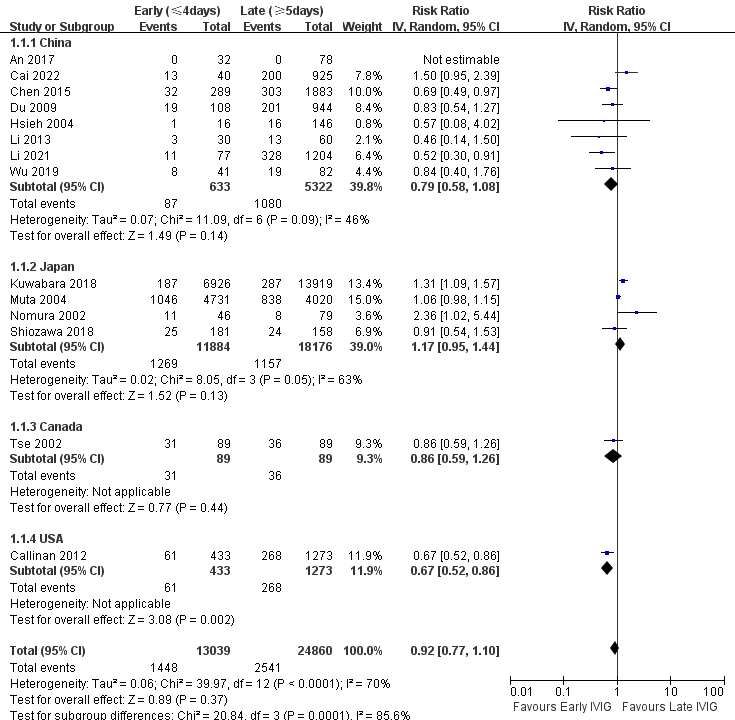
**

**Supplementary Material, Appendix S12: Subgroup analysis of CAA in the acute phase for Early IVIG VS Late IVIG.**

**
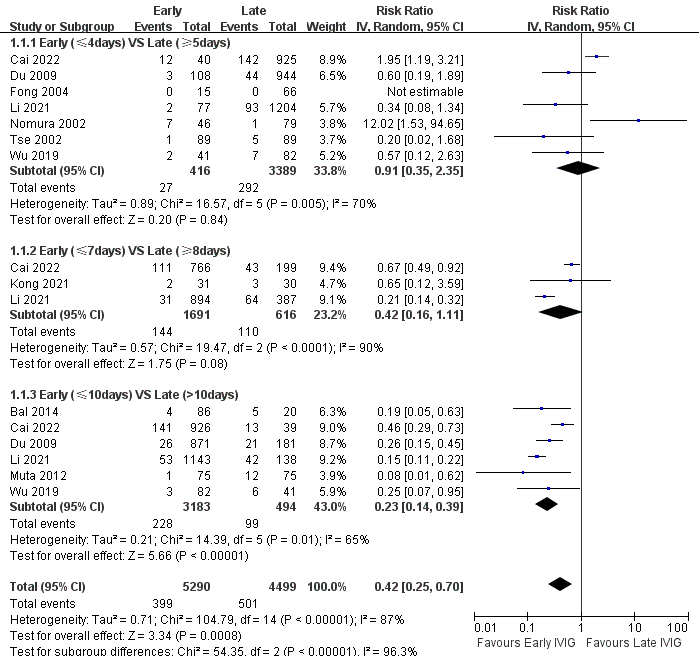
**

**Supplementary Material, Appendix S13: Subgroup analysis of CAA during 1-2 months follow-up for Early IVIG VS Late IVIG.**


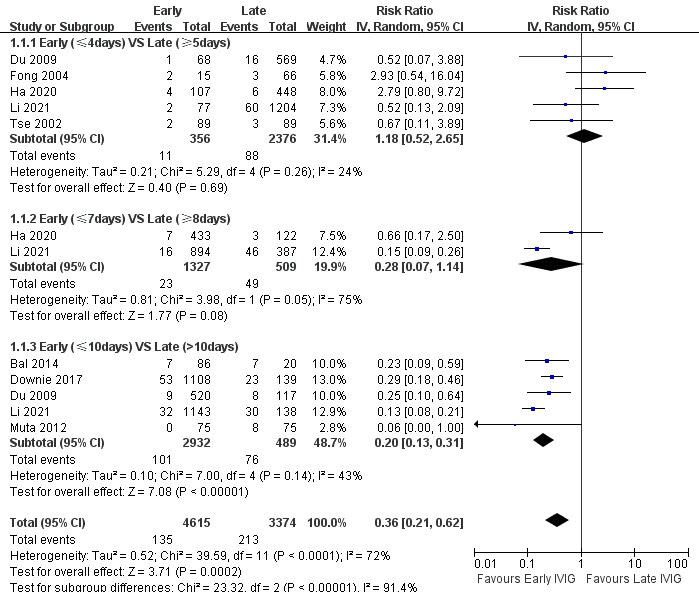


**Supplementary Material, Appendix S14: Subgroup analysis of coronary artery dilation in acute phase for Early IVIG VS Late IVIG.**

**
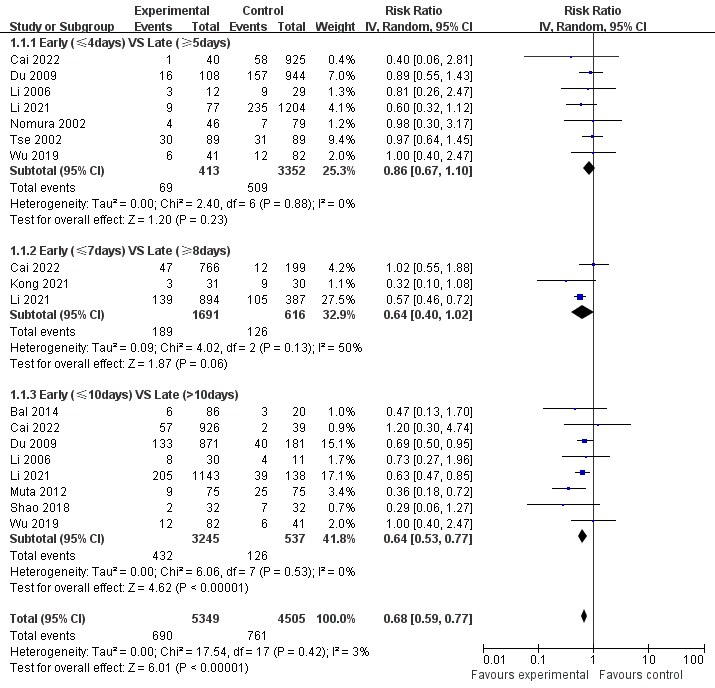
**

**Supplementary Material, Appendix S15: Subgroup analysis of coronary artery dilation during 1-2 months follow-up for Early IVIG VS Late IVIG.**


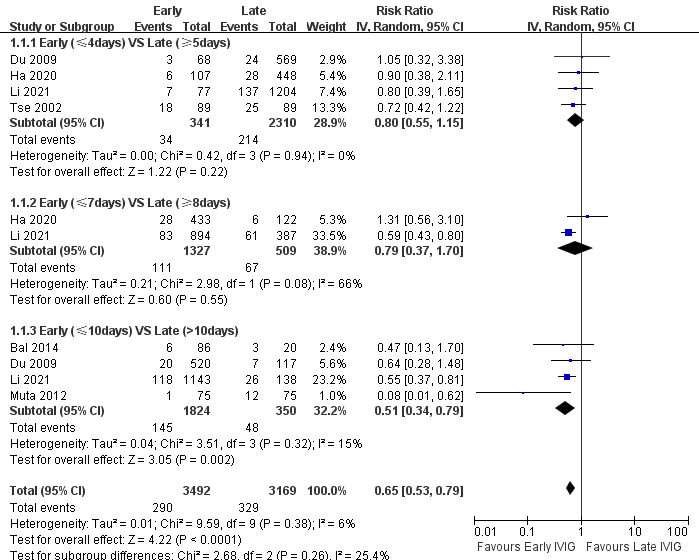


**Supplementary Material, Appendix S16: Funnel plot of initial IVIG resistance.**

**Appendix S16-A Early IVIG (≤4days) VS Late IVIG (≥5days)**

**
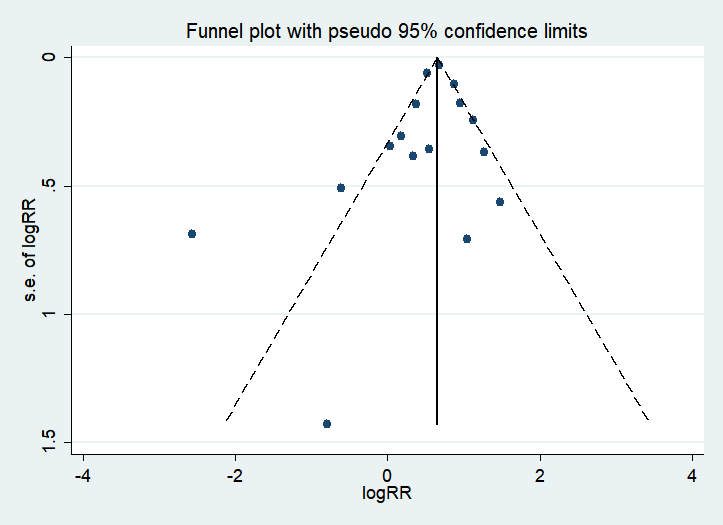
**

**Appendix S16-B Early IVIG (≤10days) VS Late IVIG (>10days)**

**
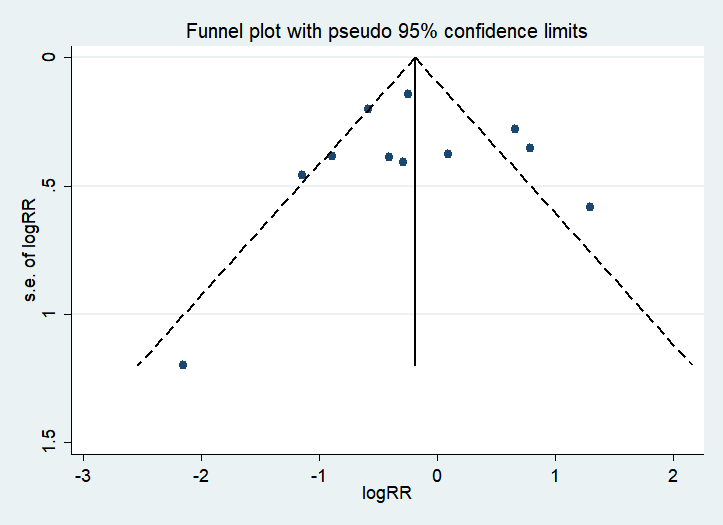
**

**Supplementary Material, Appendix S17: Funnel plot of CALs in acute phase.**

**Appendix S17-A Early IVIG (≤4days) VS Late IVIG (≥5days)**

**
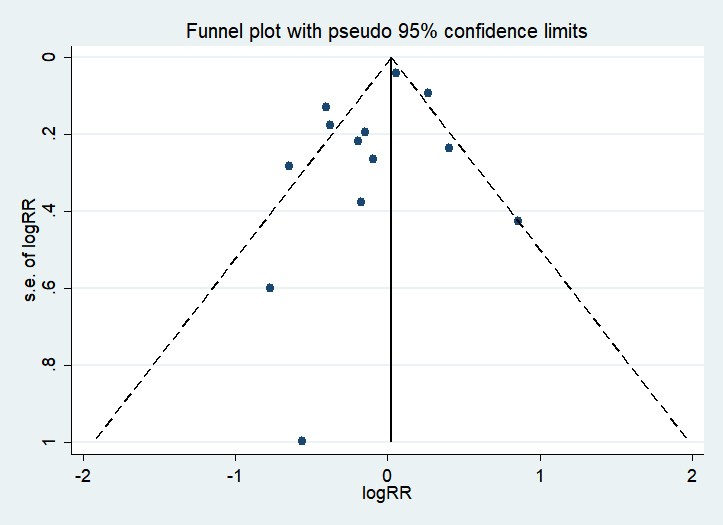
**

**Appendix S17-B Early IVIG (≤10days) VS Late IVIG (>10days)**

**
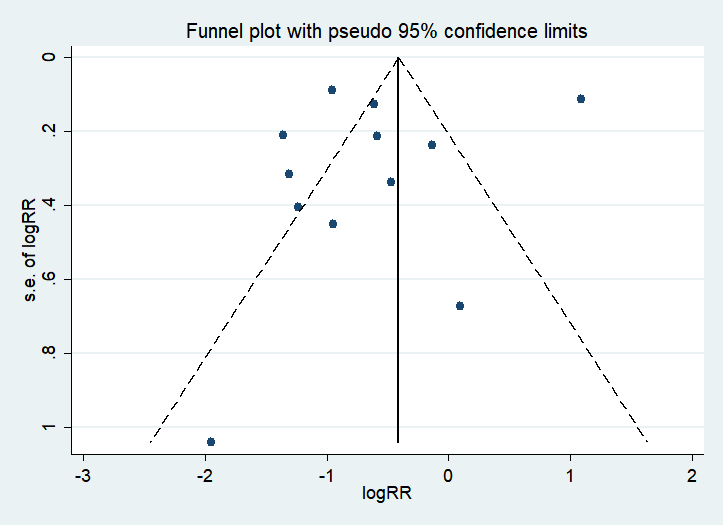
**
